# Characterization of thiol‐based redox modifications of *Brassica napus* SNF1‐related protein kinase 2.6‐2C

**DOI:** 10.1002/2211-5463.12401

**Published:** 2018-03-05

**Authors:** Tianyi Ma, Mi‐Jeong Yoo, Tong Zhang, Lihong Liu, Jin Koh, Wen‐Yuan Song, Alice C. Harmon, Wei Sha, Sixue Chen

**Affiliations:** ^1^ College of Life Sciences Northeast Forestry University Harbin China; ^2^ Department of Biology Genetics Institute University of Florida Gainesville FL USA; ^3^ College of Life Sciences, Agriculture and Forestry Qiqihar University Heilongjiang China; ^4^ Proteomics and Mass Spectrometry Interdisciplinary Center for Biotechnology Research University of Florida Gainesville FL USA; ^5^ Department of Plant Pathology University of Florida Gainesville FL USA; ^6^ Plant Molecular and Cellular Biology University of Florida Gainesville FL USA

**Keywords:** BnSnRK2.6‐2C, mass spectrometry, monobromobimane, phosphorylation, redox regulation, thiol modification

## Abstract

Sucrose nonfermenting 1‐related protein kinase 2.6 (SnRK2.6), also known as Open Stomata 1 (OST1) in *Arabidopsis thaliana*, plays a pivotal role in abscisic acid (ABA)‐mediated stomatal closure. Four *SnRK2.6* paralogs were identified in the *Brassica napus* genome in our previous work. Here we studied one of the paralogs, *BnSnRK2.6‐2C*, which was transcriptionally induced by ABA in guard cells. Recombinant BnSnRK2.6‐2C exhibited autophosphorylation activity and its phosphorylation sites were mapped. The autophosphorylation activity was inhibited by S‐nitrosoglutathione (GSNO) and by oxidized glutathione (GSSG), and the inhibition was reversed by reductants. Using monobromobimane (mBBr) labeling, we demonstrated a dose‐dependent modification of BnSnRK2.6‐2C by GSNO. Furthermore, mass spectrometry analysis revealed previously uncharacterized thiol‐based modifications including glutathionylation and sulfonic acid formation. Of the six cysteine residues in BnSnRK2.6‐2C, C159 was found to have different types of thiol modifications, suggesting its high redox sensitivity and versatility. In addition, mBBr labeling on tyrosine residues was identified. Collectively, these data provide detailed biochemical characterization of redox‐induced modifications and changes of the BnSnRK2.6‐2C activity.

**Abbreviations**
[Ca^2+^]_cyt_: cytosolic Ca^2+^ concentration
ABA: abscisic acid
CBB: Coomassie Brilliant Blue R‐250
CDS: coding DNA sequences
cysTMT: cysteine reactive tandem mass tagging
GCP: guard cell protoplasts
Grx: glutaredoxin
GSH: reduced glutathione
GSNO: S‐nitrosoglutathione
GSSG: oxidized glutathione
IAM: iodoacetamide
ICAT: isotope‐coded affinity tagging
iodoTMT: iodoacetyl tandem mass tagging
IPTG: isopropyl‐beta‐D‐thiogalactopyranoside
mBBr: monobromobimane
MeJA: methyl jasmonate
OST1: open stomata 1
PP2C: phosphatase 2C
PSM: peptide spectrum match
PTM: post‐translational modification
RCAR/PYR1/PYL: regulatory component of ABA receptor/pyrabactin resistance 1/pyr1‐like
ROS: reactive oxygen species
RSO_2_H: sulfinic acids
RSO_3_H: sulfonic acids
RSOH: sulfenic acids
SLAC1: slow anion channel associated 1
SnRK: sucrose nonfermenting 1‐related protein kinase

Crop yield depends largely on CO_2_ assimilation during photosynthesis, which is accompanied by water loss via transpiration. Both CO_2_ uptake and water loss occur mainly through stomata, the microscopic pores formed by pairs of guard cells in the leaf epidermis 1, 2, 3. Stomatal opening and closing are responsive to many environmental factors and play a critical role in response to abiotic (e.g., drought) and biotic (e.g., pathogen invasion) stresses 2. In addition, modulation of stomatal aperture by phytohormones like abscisic acid (ABA) and methyl jasmonate (MeJA) has been well studied 3, 4, 5, 6, 7, 8, 9. For example, both ABA and MeJA activate Ca^2+^ channels and S‐type anion channels, leading to an elevated cytosolic Ca^2+^ concentration ([Ca^2+^]_cyt_) and an increase in the cytosolic pH, which in turn trigger stomatal closure 8, 9. Importantly, both nitric oxide (NO) and reactive oxygen species (ROS) function as second messengers in the ABA‐ and MeJA‐regulated stomatal movement. Similar to ROS, NO regulates Ca^2+^ channels to control cytosolic Ca^2+^ concentration in guard cells 9, 10, 11, 12. The functions of NO, ROS, and other weak oxidants in guard cells have been attributed to their abilities to induce oxidative modifications including S‐nitrosylation and S‐glutathionylation of key proteins 13, 14, 15. In particular, cysteine residues are prone to such modifications due to their highly active nucleophilic sulfhydryl groups 16, making redox‐induced thiol modifications an essential process in cellular signaling 17, 18. Recently, ABA‐induced stomatal closure was found to be inhibited by S‐nitrosylation of Cys 137 in OPEN STOMATA 1 (OST1, SnRK2.6) in *Arabidopsis thaliana*, a key protein in the guard cell ABA signaling pathway 14, 19. In addition to the Arabidopsis OST1, major changes in S‐nitrosylation and S‐glutathionylation of a plethora of other proteins have been found to result from abiotic stress‐induced ROS production and/or direct oxidant treatment 20, 21. For example, a SnRK2.4 in *Brassica napus* guard cells is redox‐modified upon ABA treatment. Additionally, its activity is inhibited by H_2_O_2_, S‐nitrosoglutathione (GSNO), and oxidized glutathione (GSSG), and recovered by treatment with dithiothreitol (DTT), implying that ABA may induce reversible redox modification of SnRK2s in guard cells 22.

Models of ABA signaling with many molecular components have been proposed 6, 9, 23, 24, including the OST1 25, 26, 27, 28. OST1 belongs to a plant‐specific kinase group, sucrose nonfermenting 1‐related kinase 2 (SnRK2). Other members of the SnRK2 family such as SnRK2.2 and SnRK2.3 can also be activated by ABA, mostly in seed development and dormancy 29, 30, 31. In the absence of ABA, these SnRK2s are bound to clade A PHOSPHATASE 2Cs (PP2Cs) and thus are inhibited. In the presence of ABA, PP2Cs are bound by ABA receptors, RCAR/PYR1/PYL (REGULATORY COMPONENT OF ABA RECEPTOR/PYRABACTIN RESISTANCE 1/PYR1‐LIKE), releasing the inhibition of the SnRK2.6 29, 32, 33. Active SnRK2.6 phosphorylates an array of substrates such as bZIP transcription factors 34, 35, SLOW ANION CHANNEL ASSOCIATED 1 (SLAC1), and H^+^‐ATPases to induce stomatal closure 26, 36, 37, 38, 39, 40, 41. Similar functions of OST1 were found in maize (*Zea mays*) 42, tomato (*Solanum lycopersicum*) 43, black cottonwood (*Populus trichocarpa*) 44, and cabbage (*Brassica oleracea*) 45. Interestingly, OST1 was also required in regulating stomatal movement triggered by factors other than ABA, such as red light, yeast elicitor, and CO_2_ 46, 47, making this kinase a convergence point for various stimuli. Furthermore, OST1 also functions in plant drought response 45, 48, low temperature response 49, 50, 51, seed development and dormancy 52, and fruit ripening^53^, suggesting the versatility of the kinase and multiple regulatory mechanisms involved in the different processes.

While the physiological functions of OST1 have been extensively studied, interest in elucidation of the relationship between the kinase and ROS has emerged. It has been demonstrated that OST1 can act either upstream or downstream of ROS in the ABA signaling pathway 54, 55, 56, 57. For example, OST1 can phosphorylate AtRBOH F NADPH oxidase to boost ROS production 58; oxidant treatment of OST1 can trigger the alteration of its phosphorylation activity *in vitro* 14, indicating that the activity of OST1 could be redox‐regulated. However, the molecular mechanism underlying the redox regulation of OST1 is not fully understood. For example, it is not known whether the Cys 137 nitrosylation in Arabidopsis 14 also occurs in other OST homologs, and whether other types of redox modification play a role in the kinase regulation.

We have previously shown that transcripts of two *SnRK2.6* genes, *BnSnRK2.6‐2A* (BnaAnng41460D) and *BnSnRK2.6‐2C* (BnaC07g00000D), which are highly expressed in *B. napus* guard cells, are induced by ABA and drought 48. The two SnRK2.6 genes encode the same protein sequence, namely BnSnRK2.6‐2C. Although the effect of NO on SnRK2.6 activity has been reported in Arabidopsis 14, a comprehensive investigation of redox regulation of SnRK2.6 from any species has not been conducted. In this study, we focused on the redox regulation of the SnRK2.6‐2C from *B. napus*, an important oilseed crop. The activity of recombinant BnSnRK2.6‐2C was inhibited by GSSG and GSNO treatments, and the inhibition was reversed by reductant treatment. Different thiol modifications of BnSnRK2.6‐2C were identified including sulfonic acids and S‐glutathionylation. Interestingly, C159 was identified in all the samples and subjected to various thiol post‐translational modifications (PTMs). As C159 is the only cysteine residue in the “activation loop” of the kinase, the biological significance of the PTMs is of interest. The results reported here form the foundation for further studies of redox regulation of BnSnRK2.6‐2C and its C159 in the physiological context of stomatal functions.

## Results

### Structural analysis of BnSnRK2.6‐2C, a member of the Group III of SnRK2 family

**Figure 1:**
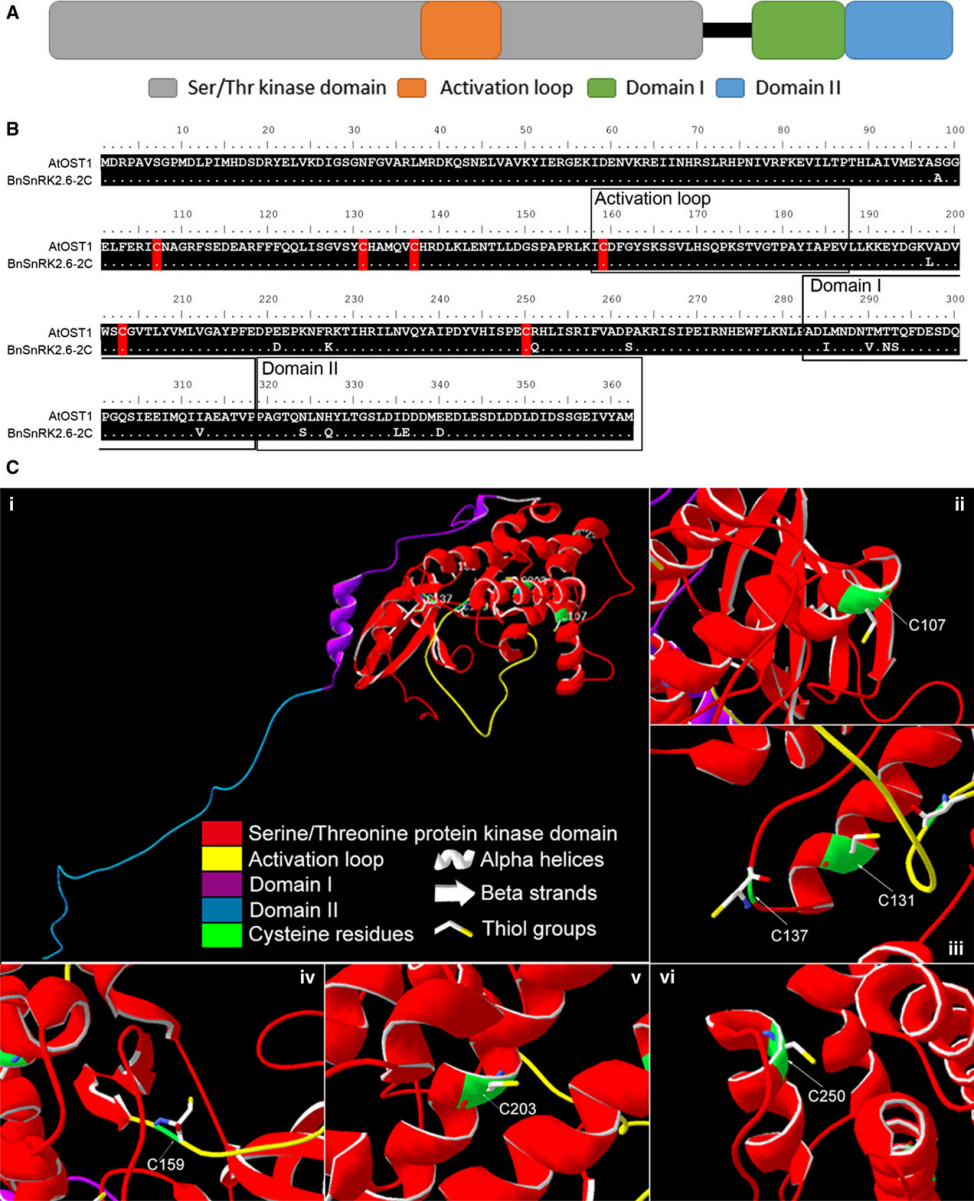
Structural analysis of BnSnRK2.6‐2C. (A) Conserved domains of BnSnRK2.6‐2C. A serine/threonine protein kinase domain with activation loop, a SnRK2‐specific domain (Domain I) and a SnRK2, group III‐specific domain (Domain II) were predicted. (B) Comparison of amino acids sequences of BnSnRK2.6‐2C and AtOST1 (At4g33950). Sequences of conserved domains are shown in boxes, and locations of cysteine residues are labeled in red. (C) Prediction of the tertiary structure of BnSnRK2.6‐2C. Different secondary structures are shown in cartoon, and domains are shown in different colors (i); cysteine residues and thiol groups are labeled in (ii) to (vi).

The coding DNA sequences (CDSs) of *BnSnRK2.6‐2C* and *BnSnRK2.6‐2A* were cloned from *B. napus* GCPs and sequenced in our previous work, and both genes were highly expressed in GCPs and induced by ABA and drought treatments 48. CDSs of the two paralogs are identical in length to their *A. thaliana* ortholog *AtOST1* (At4 g33950) (Fig. S1A). In addition, the three coding sequences show 99.2% similarity (Fig. S1A). The deduced amino acid sequences encoded by the *BnSnRK2.6‐2C* and *BnSnRK2.6‐2A* are identical, and the encoded protein was named as BnSnRK2.6‐2C because *BnSnRK2.6‐2C* showed higher expression in control and ABA‐treated *B. napus* GCPs 48. As shown in Fig. 1A, BnSnRK2.6‐2C belongs to group III of the SnRK2 kinase family with a serine/threonine protein kinase domain containing the activation loop, a SnRK2‐specific domain (Domain I) for osmotic stress response, and a group III SnRK2‐specific domain (Domain II) for ABA response 59.

Comparison of BnSnRK2.6‐2C with AtOST1 shows that the identity of the overall protein sequences and that of the conserved serine/threonine protein kinase domain were 95.6% and 99.3%, respectively, suggesting similar functions of BnSnRK2.6‐2C and AtOST1. In addition, six cysteine residues are present in both kinases, and all are located at the same positions within the serine/threonine protein kinase domain (Fig. 1B). Moreover, the location of the six cysteine residues in OST1s is highly conserved from different plant species (Fig. S1B) 42, 43, 44, 45, 53. The three‐dimensional structure of BnSnRK2.6‐2C was predicted using the RaptorX server (http://raptorx.uchicago.edu/) with the X‐ray crystal structure of AtOST1 60 as a reference. As shown in Fig. 1C‐i, two α‐helices were found in Domain I, and no secondary structure was predicted for Domain II (Note that the structure of domain II was disordered in 3UC4 and not modeled). Four cysteine residues (C107, C131, C203, and C250) are located in α‐helices in the large lobe (Fig. 1C‐ii, iii, v, vi) with C131, C203, and C250 being buried in the structure (Fig. 1C‐iii, v, vi) and C107 on the surface (Fig. 1C‐ii.). C137 and C159 are located in loops (Fig. 1C‐iii, iv). C137 is on the surface, while C159 is located in the catalytic cleft. The thiol groups of C107, C137, and C159 are predicted to face the outside of the protein (Fig. 1C‐ii, iii, iv), whereas those of C131, C203, and C250 face toward the core (Fig. 1C‐iii, v, vi). No disulfide bonds were found between the six cysteines in either the reference or the predicted structures (Fig. 1C). In the structure of the complex between OST1 with the type C protein phosphatase HAB1 (Protein Data Bank ID: 3UJG 61), the conformation appears to be different and C131 and C159 are very close together, but no disulfide bond between them is noted. C137 is nearby, but its S atom is pointed away from C131 and C159.

### Redox‐regulated autophosphorylation activity of recombinant BnSnRK2.6‐2C

**Figure 2:**
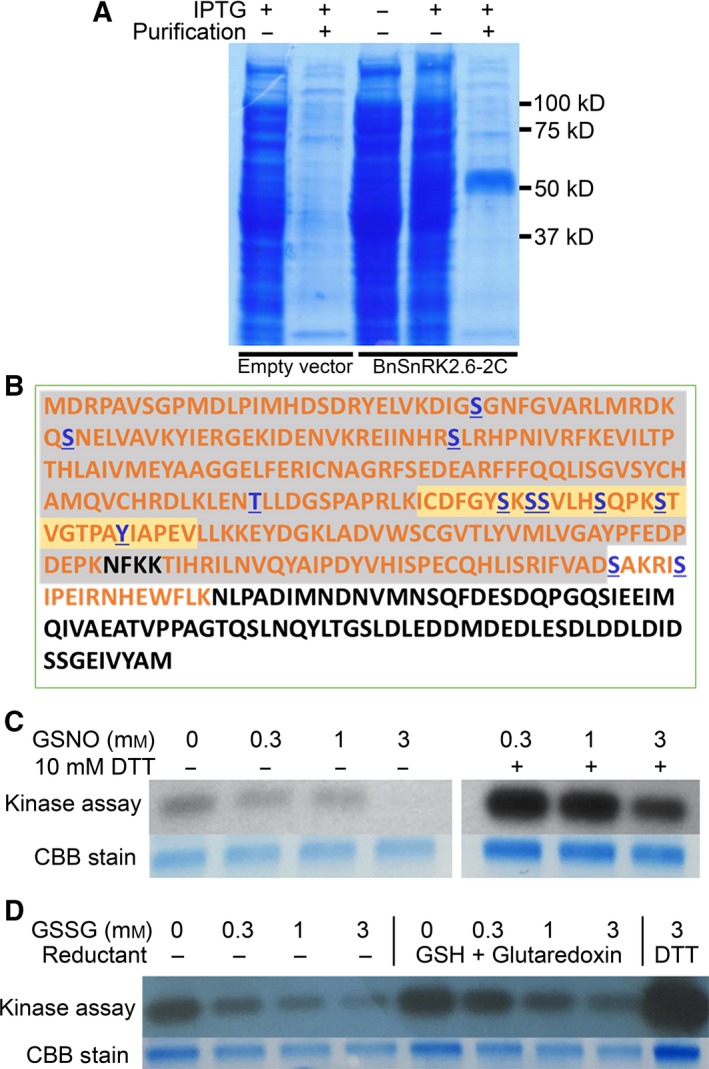
Autophosphorylation and redox regulation of recombinant BnSnRK2.6‐2C. (A) Expression and purification of recombinant BnSnRK2.6‐2C. Empty pET28a vector was used as control. (B) Peptides and phosphorylation sites of BnSnRK2.6‐2C identified by LC‐MS/MS. Sequences of peptides identified are shown in orange, and phosphorylation sites identified are shown in blue with underlines. The serine/threonine protein kinase domain is shaded in gray with the activation loop shaded in yellow. (C) Autophosphorylation activity of BnSnRK2.6‐2C treated with GSNO and/or DTT. Upper panel: autoradiograph of BnSnRK2.6‐2C. Lower panel: Coomassie Blue staining as loading control. (D) Autophosphorylation activity of BnSnRK2.6‐2C treated with GSSG and/or GSH with glutaredoxin or DTT. Upper panel: autoradiograph of BnSnRK2.6‐2C. Lower panel: Coomassie Blue staining as loading control.

Recombinant BnSnRK2.6C was purified, and a dominant band with the predicted size (~ 51 kDa) was observed on SDS/PAGE (Fig. 2A). To validate the identity of the expressed protein and to map its *in vitro* phosphorylation sites, the BnSnRK2.6C protein was subjected to an autophosphorylation reaction followed by digestion with trypsin (http://www.chem.qmul.ac.uk/iubmb/enzyme/EC3/4/21/4.html), and the resulting peptides were used for protein identification and phosphorylation site mapping by LC‐MS/MS. The identified peptides covered more than 70% of the protein sequence, with 12 phosphorylation sites identified (Fig. 2B, Fig. S2).

To investigate potential redox effects on BnSnRK2.6‐2C activity with the consideration of GSH being a main regulator of cellular redox status, two oxidized forms of glutathione, GSSG and GSNO, were used for treatment before performing kinase activity assays. As shown in Fig. 2C,D, the autophosphorylation activity of BnSnRK2.6‐2C was suppressed by GSNO and GSSG in a dose‐dependent manner. To test whether the oxidant‐induced inhibition of kinase activity is reversible, the pre‐oxidized samples were further treated either with DTT as a general reductant or with reduced glutathione (GSH) and glutaredoxin (Grx, http://www.chem.qmul.ac.uk/iubmb/enzyme/EC1/20/4/1.html) as specific reductants for S‐glutathionylation. The results showed that the kinase activity of GNSO‐ and GSSG‐oxidized BnSnRK2.6‐2C can be recovered to different extents by the reductants. Compared to the combination of GSH and Grx, DTT showed a stronger effect in enhancing the kinase activity. It was also noteworthy that BnSnRK2.6‐2C without oxidant treatment showed an increase in activity by reductant, indicating that a more reducing environment favors the active form of the kinase.

### Detection of cysteine modifications in BnSnRK2.6‐2C by mBBr fluorescence

**Figure 3:**
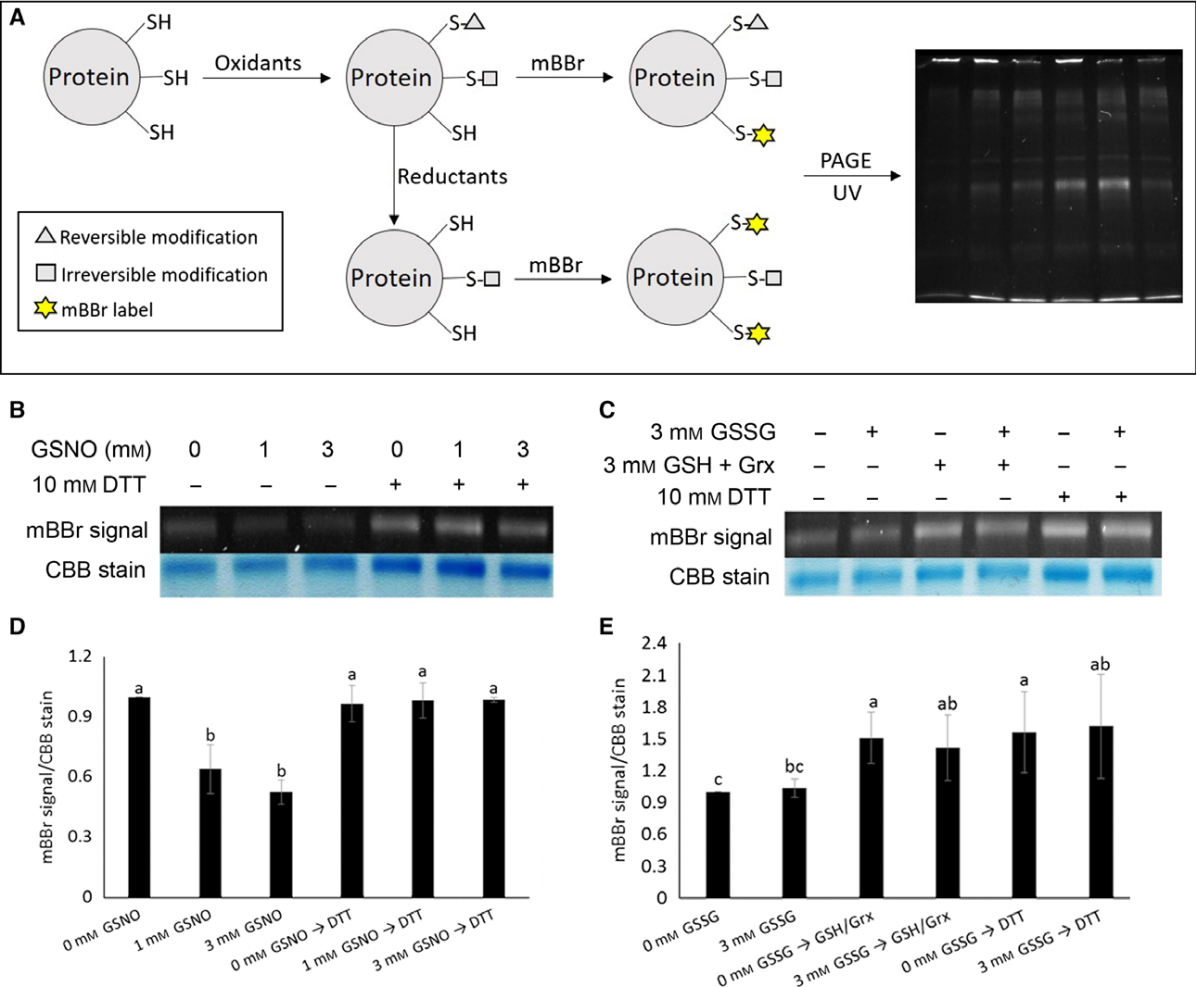
Reversible cysteine thiol oxidation occurred in BnSnRK2.6‐2C treated with GSNO or GSSG. (A) Forward mBBr labeling strategy workflow for detecting reversible cysteine thiol oxidation. Treatment of proteins with oxidants resulted in oxidation of some sulfhydryl groups (triangles or squares). Following removal of the oxidant, mBBr was used to label the free thiols (SH). Alternatively, the protein was treated with reductant, and some oxidized sulfhydryls (triangles) were returned to the reduced state (SH), while others remained blocked (squares). Subsequent treatment of protein with mBBr resulted in the modification of sulfhydryls by the reagent, and proteins with mBBr modifications appeared as fluorescent bands on UV‐illuminated gels. Reversible cysteine thiol oxidation occurred in BnSnRK2.6‐2C treated with GSNO (B) or GSSG (C). Upper panel: UV fluorescence of mBBr‐labeled BnSnRK2.6‐2C. Lower panel: Coomassie Blue staining as loading control. Relative quantification of mBBr fluorescence of BnSnRK2.6‐2C treated with GSNO (D) or GSSG (E). Fluorescence was determined by fluorescence intensity divided by the intensity of Coomassie Blue stain calculated by ImageJ software; values of 0 mm GSNO and 0 mm GSSG treatments were normalized as 1, respectively. Three replicates were used for statistical analysis, Duncan method was used in one‐way ANOVA analysis, *P* < 0.05, and standard errors were indicated.

To further confirm that GSSG‐ and GSNO‐induced modifications on cysteine residues, mBBr labeling was used to study the redox status of the thiol groups of BnSnRK2.6‐2C. A forward labeling strategy, in which free thiols available after oxidant and/or reductant treatments were labeled with mBBr directly, was followed to determine incorporation of mBBr and thus the state of free thiols (Fig. 3A). In this strategy, the intensity of the mBBr fluorescence signal under UV is inversely proportional to the number of cysteines modified by the oxidants. As inferred from the kinase activity data, application of GSNO led to a significant drop in the free thiol content of BnSnRK2.6‐2C, which was restored to the original level by DTT (Fig. 3B,D). These data suggest that GSNO causes reversible cysteine modifications. In contrast, no significant changes in mBBr labeling patterns were observed in the GSSG‐treated samples compared to untreated control (Fig. 3C,E). Initially, it was thought that the GSSG concentration used in the assays might not be high enough to reveal the differences. However, even a treatment with 24 mm GSSG did not lead to significant changes in the fluorescence signal (data not shown). Clearly, GSSG treatment caused cysteine PTMs based on the mass spectrometry results (Table 1). This result may indicate the limitation of the mBBr method used here.

**Table 1:** Overview of peptides with cysteine thiol modifications in different redox treatments. The percentage in parenthesis represents the fraction of peptide spectral matches (PSMs) of the peptides containing modified cysteine residues relative to the total PSMs of BnSnRK2.6‐2C. Positive control: The protein was reduced with DTT, labeled with mBBr directly, and then treated with IAM. Negative control: DTT‐reduced free thiols were blocked with IAM before the mBBr labeling. DTT control: The protein was blocked with IAM, then reduced with DTT and labeled with mBBr. GSH control: The protein was blocked with IAM and then reduced with GSH and Grx, followed by mBBr labeling. GSNO, GSSG, and GSSG + GSH treatments (reverse labeling): After the treatment, IAM was used to block the remaining free thiol groups. Reversibly oxidized cysteine residues were then reduced with DTT or GSH and Grx, followed by mBBr labeling

	Positive control	Negative control	DTT control	GSH control	GSNO treatment	GSSG treatment	GSSG + GSH treatment
IAM	C159 (0.21%)^a^	C107 (0.57%) C131 (0.13%) C137 (0.13%) C159 (9.19%) C203 (0.26%) C250 (0.77%)	C107 (0.25%) C159 (8.72%)	C107 (0.07%) C159 (6.88%)	C107 (0.18%) C159 (4.21%)	C159 (5.32%) C203 (0.12%)	C159 (5.75%) C250 (0.64%)
mBBr	C107 (0.53%) C159 (7.22%)		C159 (0.20%)	C159 (0.60%)	C159 (0.57%)	C131 (0.11%)^b^ C137 (0.11%)^b^ C159 (0.84%)	C131 (0.20%)^b^ C137 (0.20)^b^ C159 (0.51%)
Glutathionylation				C159 (0.27%)	C107 (0.30%) C159 (0.79%)	C159 (1.27%)	C159 (0.63%) C250 (0.12%)^b^
Sulfinic/sulfonic acid	C159 (0.07%)	C159 (0.13%)	C159 (0.50%)	C159 (0.39%)	C159 (0.38%)	C159 (0.42%)	C159 (0.17%)

a Numbers in brackets are percentages of the PSMs of peptides containing modified cysteine residues over the total PSMs of the recombinant BnSnRK2.6‐2C in different treatments.

b Indicates cysteine‐containing peptides detected by LC‐MS/MS but the quality of MS2 spectra was of relatively low confidence. All the data represent average of four replicates.

### Multiple cysteine modifications of BnSnRK2.6‐2C identified by LC‐MS/MS

To map the oxidant‐induced modifications of BnSnRK2.6‐2C, LC‐MS/MS was used to determine cysteine modifications. Because cysteine modifications including S‐nitrosylation and S‐glutathionylation may be unstable in the MS assay, a reverse labeling method was used to determine which cysteine residues were reversibly oxidized (Materials and methods, Fig. S3). In this case, the detection of mBBr labeling indicates that the cysteine‐containing peptides undergo reversible modifications caused by the oxidants. To ensure the efficiency of mBBr labeling and iodoacetamide (IAM) alkylation, a positive control with neither oxidant treatment nor IAM alkylation before mBBr labeling, and a negative control with DTT treatment before alkylation were added (Table 1, Fig. S3).

**Figure 4:**
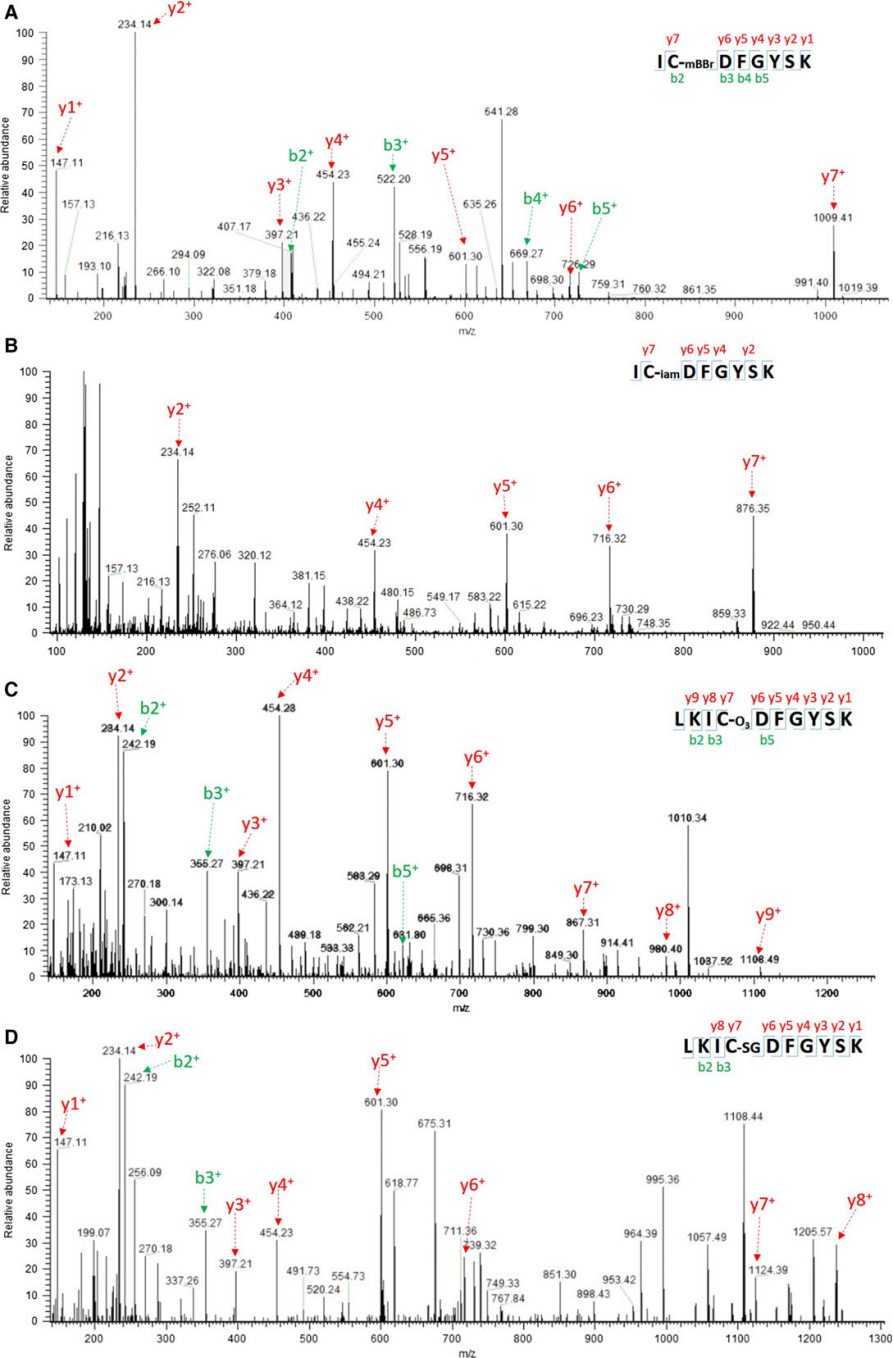
Multiple cysteine thiol modifications detected on C159 of BnSnRK2.6‐2C by LC‐MS/MS. MS/MS spectra of peptides containing C159 of BnSnRK2.6‐2C with mBBr (A), IAM (B), sulfonic acid (C), or glutathione group (D). MS/MS ions used for peptides identification were labeled.

Table 1 summarizes the identified modifications, which were labeling by IAM or mBBr; glutathionylation; and oxidation of cysteine residues to sulfinic (–SO_2_H) or sulfonic (–SO_3_H) acids. Overall, each of the five peptides (including all six cysteine residues of BnSnRK2.6‐2C) was identified at least once, revealing several previously unknown modifications of BnSnRK2.6‐2C. First, not all the cysteine residues were labeled with mBBr in the positive control. This was expected, given that some cysteine residues may have been inaccessible or oxidized before the assay (Table 1), as indicated by the enhanced kinase activity following DTT treatment (Fig. 2D). In contrast, all the cysteine residues were found to be alkylated in the negative control (with the absence of unmodified cysteine residues), demonstrating that robust reduction and alkylation occurred. The reliability of mBBr labeling and IAM alkylation was validated by manually inspecting the MS2 spectra with representative spectra shown in Fig. 4A,B, and Fig. S4. Furthermore, cysteine modifications that were previously ignored or regarded as unstable for MS analysis were successfully identified in this study. For example, oxidation of cysteine residues to sulfonic acid, an irreversible modification rarely taken into consideration, was found in all samples (Fig. 4C, Table 1, Fig. S4). Interestingly, peptides with cysteine S‐glutathionylation were directly detected by MS/MS (Fig. 4D, Fig. S4). S‐glutathionylation was previously detected in indirect labeling methods or at least after enrichment of glutathionylated peptides 62. However, nitrosylation was not found in our samples on any of the cysteine residues, a result that is in contrast to the report of this modification in AtOST1 14. Another interesting observation was that GSNO‐treated BnSnRK2.6‐2C showed S‐glutathionylation modifications on C159 and C107, which were absent in the DTT control. GSNO is known to cause protein nitrosylation, glutathionylation, or both *in vivo* 63. The reaction may depend on as‐yet undetermined features of the microenvironment surrounding the cysteine residues of BnSnRK2.6‐2C. S‐glutathionylation was also observed in the GSH control and the two GSSG‐treated samples, in which the proteins were reduced with either DTT or GSH (Table 1). Thus, these results indicate GSH can lead to cysteine S‐glutathionylation, which may not be fully reversed under certain conditions.

We also investigated the possible contribution of the location of cysteine residues to their sensitivity of modifications. Among the six cysteine residues in the protein kinase domain of BnSnRK2.6‐2C, C159 was found to be most easily modified (Table 1, Fig. 4). Strikingly, different types of modifications listed in Table 1 can be found in C159‐containing peptides. One possible explanation was that C159 was in the flexible activation loop and that the thiol group was facing outward (Fig. 1C‐vi). It was also the only cysteine residue in the activation loop and located in the catalytic cleft (Fig. 1B,C). However, C107, which resides at the surface of the protein (Fig. 1C), showed modifications under the GSNO treatment condition, but not under GSSG treatment (Table 1). The low pKa of cysteine residues is largely determined by the local electrostatic environment; for example, the presence of proximal charged residues such as lysine and arginine residues decreases the pKa 64. In spite of the scarce distribution of lysine and arginine residues surrounding the other four cysteines, each was identified in at least one sample. Among them, C131, C137, and C250 appeared to be affected by redox, while no modification was detected for C203 located close to the protein core (Fig. S4, Table 1).

### mBBr‐labeled tyrosine residues of BnSnRK2.6‐2C

**Figure 5:**
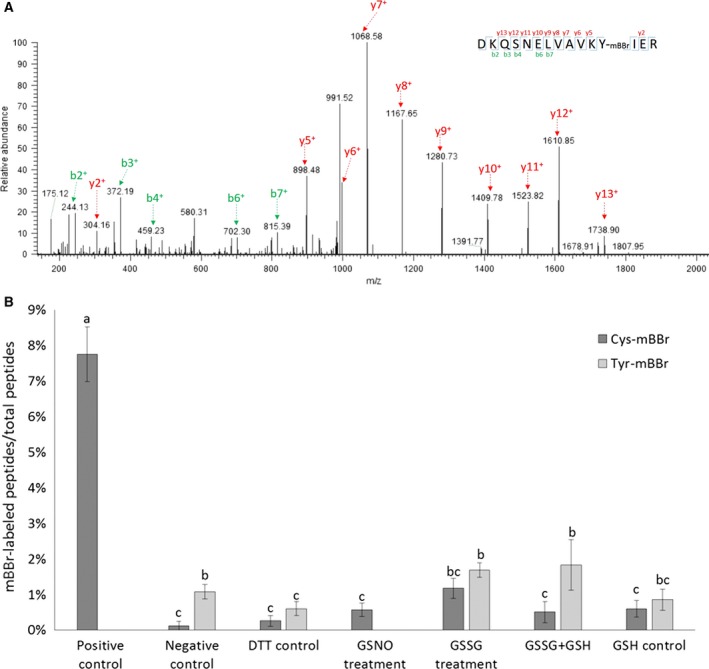
Detection of peptides containing mBBr‐labeled tyrosine residues by LC‐MS/MS. (A) MS/MS spectrum of a peptide‐containing mBBr‐labeled Y51 of BnSnRK2.6‐2C. MS/MS ions used for peptides identification were labeled. (B) Proportion of peptides with mBBr‐labeled tyrosine and cysteine residues to total peptides detected in different redox treatments. Four replicates were used for statistical analysis, Duncan method was used in one‐way ANOVA analysis, *P* < 0.05, and standard errors were indicated by error bars.

Although GSSG treatment clearly led to cysteine modifications (Table 1) and inhibited the BnSnRK2.6‐2C activity (Fig. 2D), it did not lead to significant changes in the mBBr fluorescence signal on the gel (Fig. 3E). To further understand the unchanged mBBr fluorescence, the specificity of mBBr labeling was explored. Because tyrosine residues were shown to be labeled by mBBr 65, this modification was considered in the analysis of the MS/MS data. Peptides containing mBBr‐labeled tyrosine residues Y51 or Y182 were identified with high confidence in the BnSnRK2.6‐2C (Fig. 5A, Fig. S5). Furthermore, the samples with fewer mBBr‐labeled cysteine residues seemed to have more mBBr‐labeled tyrosine residues (Fig. 5B). Since BnSnRK2.6‐2C has 11 tyrosine residues and six cysteine residues, mBBr‐labeled tyrosine could have contributed to the overall fluorescence signal of mBBr labeling. However, mBBr‐labeled tyrosine residues were not detected in the GSNO‐treated samples (Fig. 5B). Such an enigma deserves further investigation and interpretation of GSSG redox protein/proteomics data based on mBBr labeling requires caution.

## Discussion

### BnSnRK2.6‐2C is a redox‐regulated kinase

Most of the previously identified plant redox‐regulated kinases are mitogen‐activated protein kinases (MPKs) 66. For example, oxidants activate MPK1, MPK2 67, MPK3, MPK4, MPK6 68, 69, 70, and MPK12 71 in the reference plant *A. thaliana*. Similarly, activation of MPK1, MPK3, and MPK6 in *Oryza sativa* 72, 73, MPK3 and MPK5 in *Zea mays* 74, 75, and MPK1 and MPK2 in *Solanum lycopersicon* 76 and aggregation of MPK4 in *B. napus* 77 have also been observed. In contrast, other kinases including some MPKs showed decreased activity after oxidation. Recent studies showed that both SnRK2.2 and SnRK2.6 from *Arabidopsis* 14 and SnRK2.4 from *B. napus* 22 are sensitive to redox regulation, with their activities inhibited by oxidants such as GSNO and enhanced by reductants. In this study, BnSnRK2.6‐2C autophosphorylation activity was inhibited by GSNO and GSSG in a dose‐dependent manner. In addition, the inhibition could be recovered by using the general reductant DTT or specific reductants (GSH and Grx) (Fig. 2C,D). Given the availability of reactive cysteine residues in BnSnRK2.6‐2C, cysteine modifications in general were predicted to affect the kinase activity. However, certain cysteine modifications were found in both the oxidant‐ and reductant‐treated samples, in which clear differences in activity were observed (Fig. 4, Fig. S4, Table 1). Hence, it is likely that such modifications may not play a major role in inhibiting the kinase activity. For example, S‐glutathionylation on cysteine residues of BnSnRK2.6‐2C was identified in all the treated samples (albeit at different levels) in this study (Table 1), and previous studies have suggested that glutathionylation acts as a reversible protection mechanism for preventing further irreversible oxidation formation on cysteines 78. Based on the kinase activities and cysteine modifications detected in this study, it is possible that the inhibitory effect of GSSG and GSNO on BnSnRK2.6‐2C may be caused by changes in the redox microenvironment around/in the protein. Such a possibility may deserve further investigation.

### Multiple cysteine modifications occurred in the redox‐treated BnSnRK2.6‐2C

ROS or NO is known to induce redox PTMs in plant proteins 79. It is also well known that many thiol modifications are unstable and can be dynamically changed to other modifications. Protein thiols can be easily oxidized to sulfenic acids (RSOH), which serve as intermediates to other types of oxidations such as disulfides, sulfinic acids (RSO_2_H), and sulfonic acids (RSO_3_H) 80, 81, 82. Protein RSOH has also been demonstrated to be a substrate for S‐glutathionylation formation induced by GSSG and GSH 83, 84, 85. GSNO used in this study is usually considered to be a NO donor for S‐nitrosylation 14. Here we found that GSNO caused S‐glutathionylation of BnSnRK2.6‐2C, and such PTM was previously found in other proteins 79, 85, 86, but it is the first time in a SnRK2 kinase.

Given the dynamic and complicated interconversions among different cysteine modifications, we designed a multiple‐step workflow. It allowed not only to detect the final mBBr labeling, but also to monitor the changes induced by the redox treatments (Fig. 4, Fig. S4). For example, reversible cysteine oxidations of BnSnRK2.6‐2C were found in both the control and oxidant‐treated groups, suggesting that BnSnRK2.6‐2C was originally partially oxidized. In addition, the presence of mBBr in the GSH control and the absence of S‐glutathionylation in the DTT control (Table 1) indicate that the original reversible oxidation might be attributed to intra‐protein disulfide bond 86. After oxidant treatments of BnSnRK2.6‐2C, our MS‐based method also identified an increase in RSO_3_H, an important irreversible modification that was hard to detect and largely neglected in previous studies 87, 88, 89. Moreover, the identification of S‐glutathionylation with a large number of peptide spectrum matches (PSMs) in all the treatment groups and the GSH control (Fig. 4D, Fig. S4, Table 1) confirmed the contributions of GSSG, GSH, and GSNO to S‐glutathionylation formation and provided further evidence that S‐glutathionylation may not be fully reducible by Grx.

Our data confirm that the configuration of cysteine residues affects the accessibility and thus reactivity with redox reagents. For example, C159‐containing peptides were detected with abundant PSMs and diverse PTMs compared to other cysteine‐containing peptides. Interestingly, C159 is the only cysteine residue located in the flexible activation loop (Fig. 1) 60, and its location adjacent to the catalytic DFG sequence suggests this cysteine may play an important role in modulating kinase activity under different redox status. The second cysteine‐containing peptide that we observed contains C107, which resides at the outer surface. A previous study indicated that C137 in AtOST1 is modified with S‐nitrosylation 14, but it was not detected in the BnSnRK2.6‐2C (Table 1).

MS‐based proteomics approaches have been used to identify redox‐regulated thiol modifications with different cysteine‐tagging techniques such as isotope‐coded affinity tagging (ICAT) 22, 89, cysteine reactive tandem mass tagging (cysTMT) 90, 91, and iodoacetyl tandem mass tagging (iodoTMT) 92, 93. In spite of the rapid progress in discovering proteins with specific redox PTMs in response to biotic or abiotic stresses 21, 94, 95, 96, 97, only limited studies have characterized the biological functions of the redox PTMs 14, 15, 78. Different cysteine residues within a protein may occupy distinct redox microenvironment and thus be modified differently 86. It is not clear how multiple redox PTMs (e.g., those identified in this work) coordinate to bring about functional changes. Future studies on redox PTM motifs, PTM cross talk, interaction of BnSnRK2.6‐2C with redox proteins (e.g., thioredoxin and glutaredoxin), and fate of irreversibly oxidized BnSnRK2.6‐2C will help unravel the complex mechanisms of the redox regulation and its functional significance.

### mBBr is MS/MS‐compatible for redox modifications of cysteine and tyrosine residues

In this study, mBBr was chosen for indicating cysteine modification status, as well as identifying reversible thiol modifications (Figs 3 and 4, Figs S3 and S4) based on its use to specifically label cysteine thiols 98, 99, 100. It was shown that inverse relationship between mBBr fluorescence and the concentration of GSNO used in the treatment of BnSnRK2.6‐2C indicated corresponding cysteine modification levels (Fig. 3B,D). In addition, reversible thiol oxidations were detected using mBBr labeling in the MS analysis (Fig. 4A, Fig. S4). However, when using mBBr for detecting cysteine modification in GSSG‐treated BnSnRK2.6‐2C, the fluorescence showed little difference between controls and GSSG‐treated samples (Fig. 3C,E), despite cysteine modifications being identified by MS/MS in those samples (Fig. 4, Fig. S4, Table 1). Here we detected mBBr labeling of tyrosine residues, which may explain the above observation. mBBr was shown to have lower affinity to tyrosine residues than cysteine residues, and Tyr‐mBBr yields less fluorescence than Cys‐mBBr 65. Because of weaker fluorescence and reverse labeling methods, the influence of Tyr‐mBBr may not be strong on the 2D gels used in previous proteomics work 98, 99, 100. There are 11 tyrosine residues and six cysteine residues in the purified BnSnRK2.6‐2C, fluorescence of Tyr‐mBBr may influence the results of GSSG treatments (Figs 3C,E and 5B). Our detection of peptides with mBBr‐labeled tyrosine residues by MS/MS (Fig. 5A, Fig. S5) suggests that the utility of mBBr as a tyrosine label may be considered in the future.

### Summary

**Figure 6:**
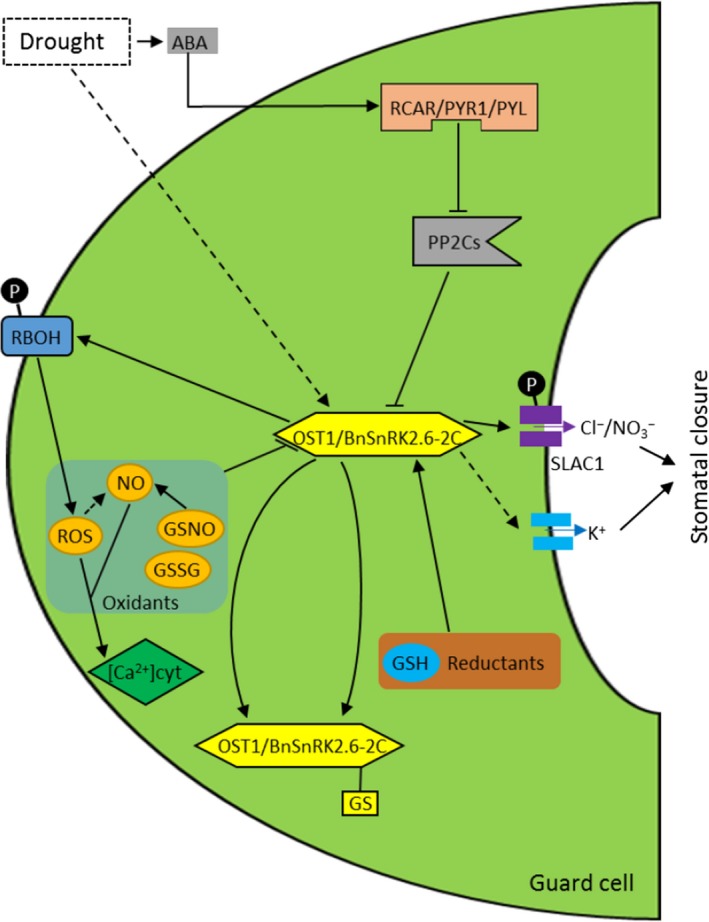
Simplified diagram depicting how redox regulation of BnSnRK2.6‐2C may affect guard cell processes leading to stomatal closure. Direct and indirect processes are indicated by solid and dashed lines, respectively.

In this study, recombinant BnSnRK2.6‐2C displayed redox‐regulated autophosphorylation activity. Oxidants GSSG and GSNO inhibited the activity of BnSnRK2.6‐2C in a dose‐dependent manner, and the inhibition was reversible. The forward labeling coupled with mBBr and reverse labeling coupled with MS analysis showed that multiple cysteine modifications including sulfonic acids and glutathionylation were formed under GSNO and GSSG treatments, and that C159 in the activation loop showed sensitivity to various modifications. mBBr can be used as a stable thiol label for evaluating cysteine redox status using LC‐MS/MS. However, our MS data also revealed that mBBr‐labeled tyrosine residues, and thus, caution is needed when interpreting the mBBr fluorescence data. Since the BnSnRK2.6‐2C shares similar amino acid sequence with AtOST1 and both can be induced by similar stimuli such as ABA or drought 48, it is highly likely that redox regulation of BnSnRK2.6‐2C may affect the guard cell processes known to be regulated by AtOST1, including cytosolic Ca^2+^ concentration changes 9, 10, 11, 12, ROS production 76, anion and potassium efflux 26, 36, 37, 38, 39, 40, 41, leading to stomatal closure (Fig. 6). This report of the different thiol modifications detected in the BnSnRK2.6‐2C will facilitate future studies of the biological implications of the redox PTMs and their cross talk with kinase phosphorylation sites and activities.

## Materials and methods

### Sequence alignment and protein structure prediction

The sequence of *OST1* from Arabidopsis (*AtOST1*) was obtained from the Arabidopsis Information Resource (TAIR, http://www.arabidopsis.org/), the cDNA of *B. napus SnRK2.6‐2A* and *SnRK2.6‐2C* were identified in our previous work 48, and OST1 sequences of other species were retrieved according to previous studies 42, 43, 44, 45, 53. Sequences were aligned by ClustalW 101, and the conserved domains were identified as previously described 59. Sequence identity was calculated using the LALIGN program (http://www.ch.embnet.org/software/LALIGN_form.html). Protein tertiary structure was predicted using the RaptorX structure prediction server (http://raptorx.uchicago.edu/) 102, and the template for modeling was downloaded from the Protein Data Bank 103. The Swiss PDB Viewer (http://spdbv.vital-it.ch) was used for analyzing the predicted protein structure.

### RNA extraction, cDNA synthesis, and gene cloning

Guard cell protoplasts (GCPs) were isolated as previously described 104. Total RNA of *B. napus* GCPs was extracted with a RNeasy® plant mini kit (Qiagen, USA) according to the manufacturer's protocol. A NanoDrop® 1000 spectrometer (Thermo Fisher Scientific Inc., USA) was used for checking RNA quality and quantity. The GoScript™ reverse transcription system (Promega, USA) was used for cDNA synthesis in a 20 μL reaction mixture using 1 μg of total RNA with random primers following the manufacturer's protocol. PCR amplification was carried out with the following primers: BnOST1.12F, 5′‐CGC*GGATCC*GCGATGGATCGACCAGCAGTG‐3′ with *Bam*H I site (italicized and underscored) and BnOST1.12R‐X, 5′‐CCG*CTCGAG*CGGCATTGCGTACACGATCTCTC‐3′ with *Xho* I site. Q5^®^ Hot Start High‐Fidelity DNA Polymerase (New England Biolabs, USA) was used for PCR product following the manufacturer's protocol. PCR products were cloned using the StrataClone Blunt PCR Cloning Kit (Stratagene, USA), and their sequences were confirmed through sequencing.

### Recombinant BnSnRK2.6‐2C expression and purification

The cloned *BnSnRK2.6‐2C* described above and the pET28a expression vector (Novagen, USA) were double‐digested with *Bam*H I‐HF and *Xho* I (New England Biolabs, USA), and the resulting gene fragment and linearized vector were ligated by T_4_ DNA Ligase (New England Biolabs, USA). The constructs were transformed into *Escherichia coli* strain BL21 (DE3) for protein expression. Positive colonies were first growing in LB medium (0.5% w/v yeast extract, 1% w/v NaCl, 1% w/v tryptone) supplemented with 50 μg·mL^−1^ kanamycin at 37 °C to OD_600_ of 0.6, and then, the expression of recombinant BnSnRK2.6‐C was induced with 1 mm isopropyl‐beta‐D‐thiogalactopyranoside (IPTG) at 37 °C for 4 h. Recombinant his‐tagged protein was purified using a Midi PrepEase® kit (Affymetrix/USB, USA) following the manufacturer's protocol. For kinase assays and mBBr labeling experiments, the recombinant BnSnRK2.6‐2C was dialyzed at 4 °C overnight in 25 mm Tris/HCl pH 7.5 containing 0.5 mm DTT and 100 μm phenylmethanesulfonyl fluoride, and then washed with a 3 kD cutoff ultra‐filtration unit (Millipore, USA). Bradford protein assay (Bio‐Rad Laboratories Inc., USA) was used for determining protein concentration with bovine serum albumin as a standard 77.

### Redox treatment and in‐solution kinase assay

The purified BnSnRK2.6‐2C protein was aliquoted into ~ 1.5 μg for each treatment. Protein aliquots were treated with 0.3, 1, and 3 mm oxidant (GSNO or GSSG) in a volume of 40 μL for 15 min, and then incubated with either 10 mm DTT or GSH and/or 1 unit of Grx (Sigma‐Aldrich Co., USA) for an additional 20 min. The amount of GSH in the treatments depended on the protein quantity, and a ratio of 1 mm GSH to 1 μg protein was used. All the treatments were performed at room temperature. Kinase activity assays were conducted as previously described 77. Briefly, 20 μL of sample was incubated with an equal volume of kinase reaction buffer (50 mm Tris/HCl, pH 7.5, 10 mm MnCl_2_, 2 μm ATP and 2 μCi [γ‐^32^P] ATP (PerkinElmer, USA)) at 30 °C for 30 min. The reaction was stopped by adding SDS/PAGE sample loading buffer, and the mixtures were incubated at 100 °C for 5 min. Samples were separated on SDS/PAGE, and the bands of interest were visualized by Coomassie Brilliant Blue R‐250 (CBB) staining as described previously 77. The activity of BnSnRK2.6‐2C in the gels was determined by autoradiography. Four independent replicates of kinase assay were used for confirming the effects caused by GSNO or GSSG.

### Cysteine modification analysis using mBBr labeling

Aliquots of 3 μg recombinant BnSnRK2.6‐2C were treated with 1 mm or 3 mm GSNO or 3 mm GSSG and reductants as described above. Redox‐treated proteins were labeled with 0.2 mg of mBBr (Thermo Fisher Scientific Inc., USA) dissolved in acetonitrile in the dark at room temperature for 1 h. Excess mBBr was removed by dichloromethane extraction. After addition of 200 μL dichloromethane, each sample was vortexed and centrifuged at 11 956 ***g*** for 2 min, and the dichloromethane in the lower phase was removed. This was repeated three times. IAM at a final concentration of 100 mm was used for blocking the remaining free thiol groups at 37 °C for 1 h in the dark. Proteins were separated on 12% SDS/PAGEs, and the gels were first washed with trichloroacetic acid for 1 h, and then washed with 10% acetic acid (v/v) and 40% methanol (v/v) overnight 105. The labeling of mBBr was determined by imaging the gel under UV light 106, and the gels were then stained with CBB to estimate the protein amount. ImageJ (https://imagej.nih.gov/ij/index.html) was used for image analysis, and the relative fluorescence signals from mBBr labeling were normalized to the protein amount indicated by CBB staining. Three independent replicates were used for statistical analysis, and the Duncan method was used for one‐way ANOVA analysis, with *P* < 0.05 as a significance cutoff.

### LC‐MS/MS identification of cysteine modifications

A reverse labeling strategy for BnSnRK2.6‐2C cysteine modification analysis is detailed in Fig. S3. Aliquots of BnSnRK2.6‐2C (~ 3 μg) were incubated with 3 mm GSSG or GSNO for 30 min to react with responsive free thiol groups, and the remaining free thiols were subsequently alkylated with 100 mm IAM. Next, the samples were treated for 30 min with DTT (GSNO‐ and GSSG‐treated samples) or GSH together with Grx (GSSG‐treated samples), followed by labeling with mBBr as described above. To test the validity of the labeling workflow, the protein aliquots were initially fully reduced with DTT and were then labeled with mBBr directly as a positive control. For the negative control, blocking of the DTT‐reduced free thiols with IAM was performed before the mBBr labeling. Another two controls consisted of an IAM blocking step, a reducing step with either DTT or GSH and Grx, and an mBBr labeling step, for testing effects of reductants only. Excessive mBBr was removed by the dichloromethane extraction described above, and the samples were digested with a modified trypsin (1 : 1 w/w) (Promega, USA) at 37 °C for 12 h. The digestion was terminated by adding 0.1% formic acid, and the tryptic peptides were then lyophilized 93. The resulting samples were cleaned up by solid‐phase extraction with ZipTip (Millipore, USA) according to the manufacturer's instructions.

The peptides were dissolved in 0.1% formic acid and then subjected to LC‐MS/MS analysis. The scan range of the precursor ions on the Q Exactive Plus (Thermo Fisher Scientific Inc., USA) was set as 400–2000 m/z, and a top 20 data‐dependent acquisition method was used for MS/MS 93. The acquired MS/MS spectra were searched against a *B. napus* database (http://www.genoscope.cns.fr/brassicanapus/data/) with an additional sequence of the recombinant BnSnRK2.6‐2C using Mascot (v2.4, Matrix Science, UK). The following dynamic modifications on the indicated amino acid residues were added: phosphorylation (STY), mBBr (CY), carbamidomethyl (C), nitrosylation (C), glutathionylation (C), oxidation (M, C), and formation of sulfinic acid and sulfonic acid (C). Other parameters were set as previously described 107. To quantify the abundance of peptides with various modifications, the number of PSMs for identifying the modified peptides was calculated. These numbers were divided by the total PSMs for all the peptides of BnSnRK2.6‐2C to normalize the relative abundance across different samples.

## Author contributions

SC, MJY, and TZ planned the experiments; TM, MJY, LL, and WYS performed the experiments; TM, ACH, JK, and WS analyzed data; all the authors contributed to the writing of the manuscript, and ACH and SC finalized the manuscript.

## Supporting information


**Fig. S1.** Alignment of BnSnRK2.6‐2C sequence with other OST1 homologs. (A) Sequence alignment of cDNA sequences of *BnSnRK2.6‐2A*,* BnSnRK2.6‐2C* and *AtOST1*. (B) Sequence alignment of amino acid sequences of functionally studied OST1s of different plant species. OST1 sequences of *Brassica oleracea* (BolOST1‐1, GenBank ID: AHE78413.1), *Fragaria vesca* subsp. vesca (FaSnRK2.6, NCBI reference sequence: XP_004290308.1), *Solanum lycopersicum* (SolycOST1, NCBI reference sequence: XP_004230794.1), *Populus trichocarpa* (PtSnRK2.11, NCBI reference sequence: XP_002313835.1; PtSnRK2.12, NCBI reference sequence: XP_006384459.1) and *Zea mays* (ZmOST1, GenBank ID: ACG36261.1) were used for alignment together with AtOST1 and BnSnRK2.6‐2C. The locations of cysteine residues are labeled with red boxes.

**Fig. S2.** Phosphorylation sites identification of BnSnRK2.6‐2C by mass spectrometry. MS/MS spectra of peptides containing phosphorylated S29 (A), S43 (B), S71 (C), T146 (D), S164 (E), S166 (F), S167 and S171 (G), S175 (H,I), Y182 (I), S262 (J), and S267 (K) of BnSnRK2.6‐2C detected by LC‐MS/MS. MS/MS ions used for peptides identification were labeled.

**Fig. S3.** Diagram depicting monobromobimane (mBBr) labeling workflow to identify reversible oxidations of cysteine residues in BnSnRK2.6‐2C and the positive and negative controls (Table1for results). Positive control: The protein was reduced with DTT, labeled with mBBr directly, and then treated with IAM. Negative control: DTT‐reduced free thiols were blocked with IAM before the mBBr labeling. DTT control: The protein was blocked with IAM, then reduced with DTT and labeled with mBBr. GSH control: The protein was blocked with IAM, and then reduced with GSH and Grx, followed by mBBr labeling. GSNO, GSSG and GSSG + GSH treatments (reverse labeling): After the treatment, IAM was used to block the remaining free thiol groups. Reversibly oxidized cysteine residues were then reduced with DTT or GSH and Grx, followed by mBBr labeling.

**Fig. S4.** Identification of BnSnRK2.6‐2C cysteine residue modifications in response to GSNO and GSSG treatments by mass spectrometry. MS/MS spectra of peptides containing C107 modified by IAM (A), mBBr (B), or glutathione group (C), C131 and C137 modified by IAM (D), C203 modified by IAM (E), C250 modified by IAM (F) or mBBr (G) in BnSnRK2.6‐2C by LC‐MS/MS. MS/MS ions used for peptides identification were labeled.

**Fig. S5.** Identification of peptides containing mBBr‐labeled Y182 in BnSnRK2.6‐2C. MS/MS ions used for peptides identification were labeled.
