# Supplementary material for: Characterization of thiol‐based redox modifications of Brassica napus SNF1‐related protein kinase 2.6‐2C
Source: FEBS Open Bio. 2018 Mar 5;8(4):628–45. doi: 10.1002/2211-5463.12401 (PMC5881534; doi:10.1002/2211-5463.12401)
Supplement: Supplementary file 1 — Fig. S1. Alignment of BnSnRK2.6‐2C sequence with other OST1 homologs. (A) Sequence alignment of cDNA sequences of BnSnRK2.6‐2A, BnSnRK2.6‐2C and AtOST1. (B) Sequence alignment of amino acid sequences of functionally studied OST1s of different plant species. OST1 sequences of Brassica oleracea (BolOST1‐1, GenBank ID: AHE78413.1), Fragaria vesca subsp. vesca (FaSnRK2.6, NCBI reference sequence: XP_004290308.1), Solanum lycopersicum (SolycOST1, NCBI reference sequence: XP_004230794.1), Populus trichocarpa (PtSnRK2.11, NCBI reference sequence: XP_002313835.1; PtSnRK2.12, NCBI reference sequence: XP_006384459.1) and Zea mays (ZmOST1, GenBank ID: ACG36261.1) were used for alignment together with AtOST1 and BnSnRK2.6‐2C. The locations of cysteine residues are labeled with red boxes. [file FEB4-8-628-s001.pdf]

A

```
                     10        20        30        40        50        60        70        80        90       100
AtSnRK2.6    ATGGATCGACCAGCAGTGAGTGGTCCAATGGATTTGCCGATTATGCACGATAGTGATAGGTATGAACTCGTCAAGGATATTGGCTCCGGTAATTTTGGAG
BnSnRK2.6-2A ATGGACCGACCAGCAGTGAGTGGACCAATGGATTTGCCGATCATGCACGACAGCGATAGGTACGAGCTCGTCAAAGATATTGGCTCCGGTAACTTCGGAG
BnSnRK2.6-2C ATGGATCGACCAGCAGTGAGTGGACCAATGGATTTGCCGATCATGCACGATAGCGATAGGTACGAGCTCGTCAAAGATATTGGCTCCGGTAACTTCGGAG

                    110       120       130       140       150       160       170       180       190       200
AtSnRK2.6    TTGCGAGATTGATGAGAGACAAGCAAAGTAATGAGCTTGTTGCTGTTAAATATATCGAGAGAGGTGAGAAGATAGATGAAAATGTAAAAAGGGAGATAAT
BnSnRK2.6-2A TTGCGAGGTTAATGAGAGACAAGCAGAGTAACGAGCTTGTTGCTGTCAAATATATCGAGCGAGGTGAGAAGATAGATGAAAATGTGAAAAGGGAGATAAT
BnSnRK2.6-2C TTGCGAGGTTAATGAGAGACAAGCAGAGTAACGAGCTTGTTGCTGTCAAATATATCGAGCGAGGTGAGAAGATAGATGAAAATGTGAAAAGGGAGATAAT

                    210       220       230       240       250       260       270       280       290       300
AtSnRK2.6    CAACCACAGGTCCTTAAGACATCCCAATATCGTTAGATTCAAAGAGGTTATATTAACACCAACCCATTTAGCCATTGTTATGGAATATGCATCTGGAGGA
BnSnRK2.6-2A TAATCACAGGTCCTTAAGACATCCAAATATCGTCAGATTTAAAGAGGTCATATTAACACCAACCCATTTGGCTATTGTTATGGAATACGCAGCTGGAGGA
BnSnRK2.6-2C TAATCATAGGTCCTTAAGACATCCAAATATCGTCAGATTTAAAGAGGTCATATTAACACCAACCCATTTGGCCATTGTTATGGAATATGCAGCTGGAGGA

                    310       320       330       340       350       360       370       380       390       400
AtSnRK2.6    GAACTTTTCGAGCGAATCTGCAATGCAGGCCGCTTCAGCGAAGACGAGGCGAGGTTTTTCTTCCAGCAACTCATTTCAGGAGTTAGTTACTGTCATGCTA
BnSnRK2.6-2A GAACTCTTTGAGCGTATCTGCAATGCAGGCCGGTTCAGCGAAGACGAGGCAAGGTTTTTCTTCCAGCAACTCATTTCAGGAGTTAGTTACTGTCATGCTA
BnSnRK2.6-2C GAACTCTTTGAGCGTATCTGCAATGCAGGACGGTTCAGCGAAGACGAGGCAAGGTTTTTCTTCCAGCAACTCATTTCAGGAGTTAGTTACTGTCATGCTA

                    410       420       430       440       450       460       470       480       490       500
AtSnRK2.6    TGCAAGTATGTCACCGAGACTTAAAGCTCGAGAATACGTTATTAGATGGTAGCCCGGCCCCTCGTCTAAAGATATGTGATTTCGGAATATTCTAAGTCATC
BnSnRK2.6-2A TGCAAGTATGTCACCGAGATTTGAAGCTGGAGAATACGTTACTAGATGGTAGCCCTGCACCTCGTTTAAAGATATGTGATTTTGGTTATTCCAAGTCCTC
BnSnRK2.6-2C TGCAAGTATGTCACCGAGATTTGAAGCTGGAGAATACGTTACTAGATGGTAGCCCTGCACCTCGTTTAAAGATATGTGATTTTGGTTATTCCAAGTCCTC

                    510       520       530       540       550       560       570       580       590       600
AtSnRK2.6    AGTGTTACATTCGCAACCAAAATCAACTGTTGGAACTCCTGCTTACATCGCTCCTGAGGTTTTACTAAAGAAAGAATATGATGGAAAGGTTGCAGATGTT
BnSnRK2.6-2A AGTGTTACATTCACAGCCAAAATCAACTGTTGGAACCCCTGCTTATATCGCTCCAGAGGTTTTGCTAAAGAAAGAATATGATGGCAAGCTTGCAGATGTT
BnSnRK2.6-2C AGTGTTACATTCACAGCCAAAATCAACTGTTGGAACCCCTGCTTATATCGCTCCAGAGGTTTTGCTAAAGAAAGAATATGATGGCAAGCTTGCAGATGTT

                    610       620       630       640       650       660       670       680       690       700
AtSnRK2.6    TGGTCTTGTGGGGTTACTCTGTATGTCATGCTGGTTGGAGCATATCCTTTCGAAGATCCCGAGGAACCAAAGAATTTCAGGAAAACTATACATAGAATCC
BnSnRK2.6-2A TGGTCTTGTGGGGTAACTCTGTATGTGATGCTTGTTGGTGCATACCCTTTTGAAGATCCTGATGAGCCTAAGAATTTCAAGAAAACAATACATAGAATCT
BnSnRK2.6-2C TGGTCTTGTGGGGTAACTCTGTATGTGATGCTTGTTGGTGCATACCCTTTTGAAGATCCTGATGAGCCTAAGAATTTCAAGAAAACAATACATAGAATCT

                    710       720       730       740       750       760       770       780       790       800
AtSnRK2.6    TGAATGTTCAGTATGCTATTCCGGATTATGTTCACATATCTCCTGAATGTCGCCATTTGATCTCCAGAATATTTGTTGCTGACCCTGCAAAGAGGATATC
BnSnRK2.6-2A TGAATGTTCAGTACGCAATTCCAGATTATGTTCACATATCTCCTGAATGTCAACATCTGATCTCCAGAATATTCGTTGCTGATTCTGCAAAGAGGATATC
BnSnRK2.6-2C TGAATGTTCAGTACGCAATTCCAGATTATGTTCACATATCTCCTGAATGTCAACATCTGATCTCCAGAATATTCGTTGCTGATTCTGCAAAGAGGATTTC

                    810       820       830       840       850       860       870       880       890       900
AtSnRK2.6    AATTCCTGAAATAAGGAACCATGAATGGTTTCTAAAGAATCTACCGGCAGATCTAATGAACGATAACACGATGACCACTCAGTTTGATGAATCGGATCAA
BnSnRK2.6-2A AATTCCTGAGATAAGGAACCATGAATGGTTTCTCAAGAATCTACCAGCAGATATCATGAACGATAATGTGATGAATAGCCAGTTTGATGAATCGGACCAA
BnSnRK2.6-2C AATTCCTGAGATAAGGAACCATGAATGGTTTCTCAAGAATCTACCAGCAGATATCATGAACGATAACGTGATGAATAGCCAGTTTGATGAATCGGACCAA

                    910       920       930       940       950       960       970       980       990      1000
AtSnRK2.6    CCGGCCCAAAGCATAGAAGAAATTATGCAGATCATTGCAGAAGCAACTGTTCCTCCTGCAGGCACTCAGAATCTGAACCATTACCTCACAGGAAGCTTGG
BnSnRK2.6-2A CCAGGTCAAAGCATTGAAGAGATCATGCAGATTGTTGCAGAAGCGACTGTTCCTCCCGCAGGCACTCAGAGTCTGAACCAGTATCTCACAGGAAGCTTGG
BnSnRK2.6-2C CCAGGTCAAAGCATTGAAGAGATCATGCAGATTGTTGCAGAAGCGACTGTTCCTCCCGCAGGCACTCAGAGTCTGAACCAGTATCTCACAGGAAGCTTGG

                   1010      1020      1030      1040      1050      1060      1070      1080
AtSnRK2.6    ACATAGATGACGATATGGAGGAAGACTTAGAGAGCGACCTTGATGATCTTGACATCGACAGTAGCGGAGAGATTGTGTACGCAATGTGA
BnSnRK2.6-2A ATTTAGAAGATGATATGGATGAAGATCTAGAGAGCGACTTGGATGATCTTGACATCGACAGTAGCGGAGAGATTGTGTACGCAATGTGA
BnSnRK2.6-2C ATTTAGAAGATGATATGGATGAAGATCTAGAGAGCGACTTGGATGATCTTGACATCGACAGTAGCGGAGAGATCGTGTACGCAATGTGA
```

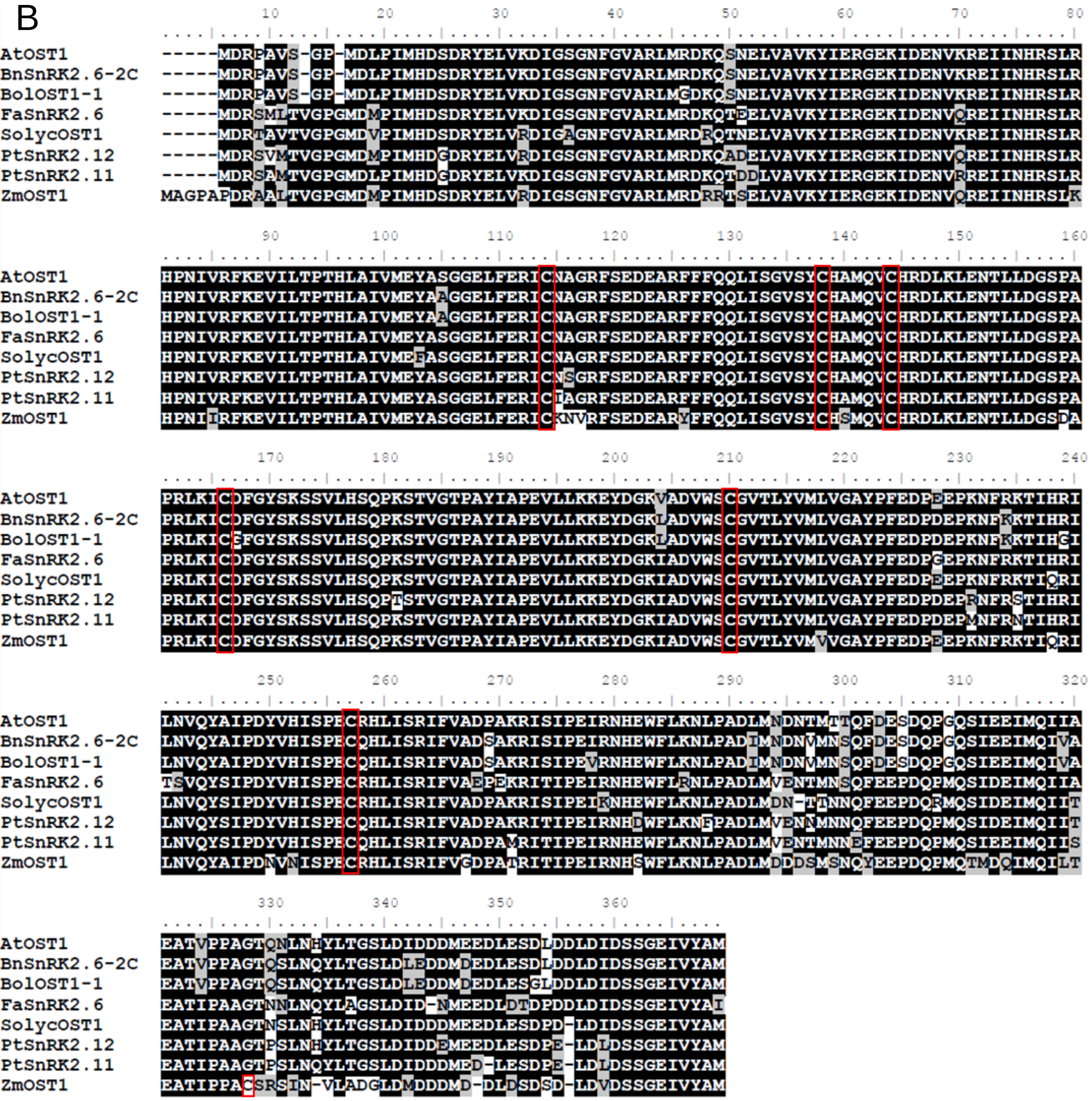
B
AtOST1 -----MDRPAVS-GP-MDLPIMHDSDRYELVKDIGSGNFGVARLMRDKQSNELVAVKYIERGEKIDENVKREIINHRSLR
BnSnRK2.6-2C -----MDRPAVS-GP-MDLPIMHDSDRYELVKDIGSGNFGVARLMRDKQSNELVAVKYIERGEKIDENVKREIINHRSLR
BolOST1-1 -----MDRPAVS-GP-MDLPIMHDSDRYELVKDIGSGNFGVARLMGDKQSNELVAVKYIERGEKIDENVKREIINHRSLR
FaSnRK2.6 -----MDRSMLTVGPGMDMPIMHDSDRYELVKDIGSGNFGVARLMRDKQTEELVAVKYIERGEKIDENVQREIINHRSLR
SolycOST1 -----MDRTAVTVGPGMDVPIMHDSDRYELVRDIGAGNFGVARLMRDRQTNELVAVKYIERGEKIDENVKREIINHRSLR
PtSnRK2.12 -----MDRSVMTVGPGMDMPIMHDGDRYELVRDIGSGNFGVARLMRDKQADELVAVKYIERGEKIDENVQREIINHRSLR
PtSnRK2.11 -----MDRSAMTVGPGMDLPIMHDGDRYELVKDIGSGNFGVARLMRDKQTDDLVAVKYIERGEKIDENVRREIINHRSLR
ZmOST1 MAGPAPDRAALTVGPGMDMPIMHDSDRYELVRDIGSGNFGVARLMRDRRTSELVAVKYIERGEKIDENVQREIINHRSLK
AtOST1 HPNIVRFKEVILTPTHLAIVMEYASGGELFERICNAGRFSEDEARFFFQQLISGVSYCHAMQVCHRDLKLENTLLDGSPA
BnSnRK2.6-2C HPNIVRFKEVILTPTHLAIVMEYAAGGELFERICNAGRFSEDEARFFFQQLISGVSYCHAMQVCHRDLKLENTLLDGSPA
BolOST1-1 HPNIVRFKEVILTPTHLAIVMEYAAGGELFERICNAGRFSEDEARFFFQQLISGVSYCHAMQVCHRDLKLENTLLDGSPA
FaSnRK2.6 HPNIVRFKEVILTPTHLAIVMEYASGGELFERICNAGRFSEDEARFFFQQLISGVSYCHAMQVCHRDLKLENTLLDGSPA
SolycOST1 HPNIVRFKEVILTPTHLAIVMEFASGGELFERICNAGRFSEDEARFFFQQLISGVSYCHAMQVCHRDLKLENTLLDGSPA
PtSnRK2.12 HPNIVRFKEVILTPTHLAIVMEYASGGELFERICNSGRFSEDEARFFFQQLISGVSYCHAMQVCHRDLKLENTLLDGSPA
PtSnRK2.11 HPNIVRFKEVILTPTHLAIVMEYASGGELFERICIAGRFSEDEARFFFQQLISGVSYCHAMQVCHRDLKLENTLLDGSPA
ZmOST1 HPNIIRFKEVILTPTHLAIVMEYASGGELFERICKNVRFSEDEARYFFQQLISGVSYCHSMQVCHRDLKLENTLLDGSDA
AtOST1 PRLKICDFGYSKSSVLHSQPKSTVGTPAYIAPEVLLKKEYDGKVADVWSCGVTLYVMLVGAYPFEDPEEPKNFRKTIHRI
BnSnRK2.6-2C PRLKICDFGYSKSSVLHSQPKSTVGTPAYIAPEVLLKKEYDGKLADVWSCGVTLYVMLVGAYPFEDPDEPKNFKKTIHRI
BolOST1-1 PRLKICEFGYSKSSVLHSQPKSTVGTPAYIAPEVLLKKEYDGKLADVWSCGVTLYVMLVGAYPFEDPDEPKNFKKTIHGI
FaSnRK2.6 PRLKICDFGYSKSSVLHSQPKSTVGTPAYIAPEVLLKKEYDGKIADVWSCGVTLYVMLVGAYPFEDPGEPKNFRKTIHRI
SolycOST1 PRLKICDFGYSKSSVLHSQPKSTVGTPAYIAPEVLLKKEYDGKIADVWSCGVTLYVMLVGAYPFEDPEEPKNFRKTIQRI
PtSnRK2.12 PRLKICDFGYSKSSVLHSQPTSTVGTPAYIAPEVLLKKEYDGKIADVWSCGVTLYVMLVGAYPFEDPDEPRNFRSTIHRI
PtSnRK2.11 PRLKICDFGYSKSSVLHSQPKSTVGTPAYIAPEVLLKKEYDGKIADVWSCGVTLYVMLVGAYPFEDPDEPMNFRNTIHRI
ZmOST1 PRLKICDFGYSKSSVLHSQPKSTVGTPAYIAPEVLLKKEYDGKIADVWSCGVTLYVMVVGAYPFEDPEEPKNFRKTIQRI
AtOST1 LNVQYAIPDYVHISPECRHLISRIFVADPAKRISIPEIRNHEWFLKNLPADLMNDNTMTTQFDESDQPGQSIEEIMQIIA
BnSnRK2.6-2C LNVQYAIPDYVHISPECQHLISRIFVADSAKRISIPEIRNHEWFLKNLPADIMNDNVMNSQFDESDQPGQSIEEIMQIVA
BolOST1-1 LNVQYAIPDYVHISPECQHLISRIFVADSAKRISIPEVRNHEWFLKNLPADIMNDNVMNSQFDESDQPGQSIEEIMQIVA
FaSnRK2.6 TSVQYSIPDYVHISPECRHLISRIFVAEPEKRITIPEIRNHEWFLRNLPADLMVENTMNSQFEEPDQPMQSIDEIMQIIA
SolycOST1 LNVQYSIPDYVHISPECRHLISRIFVADPAKRISIPEIKNHEWFLKNLPADLMDN-TTNNQFEEPDQRMQSIDEIMQIIT
PtSnRK2.12 LNVQYSIPDYVHISPECQHLISRIFVADPAKRITIPEIRNHDWFLKNFPADLMVENNMNNQFEEPDQPMQSIDEIMQIIT
PtSnRK2.11 LNVQYSIPDYVHISPECQHLISRIFVADPAMRITIPEIRNHEWFLKNLPADLMVENTMNNEFEEPDQPMQSIEEIMQIIS
ZmOST1 LNVQYAIPDNVNISPECRHLISRIFVGDPATRITIPEIRNHSWFLKNLPADLMDDDSMSNQYEEPDQPMQTMDQIMQILT
AtOST1 EATVPPAGTQNLNHYLTGSLDIDDDMEEDLESDLDDLDIDSSGEIVYAM
BnSnRK2.6-2C EATVPPAGTQSLNQYLTGSLDLEDDMDEDLESDLDDLDIDSSGEIVYAM
BolOST1-1 EATVPPAGTQSLNQYLTGSLDLEDDMDEDLESGLDDLDIDSSGEIVYAM
FaSnRK2.6 EATIPAAGTNNLNQYLAGSLDID-NMEEDLDTDPDDLDIDSSGEIVYAI
SolycOST1 EATIPAAGTNSLNHYLTGSLDIDDDMEEDLESDPD-LDIDSSGEIVYAM
PtSnRK2.12 EATIPAAGTPSLNHYLTGSLDIDDEMEEDLESDPE-LDLDSSGEIVYAM
PtSnRK2.11 EATIPAAGTPSLNQYLTGSLDIDDDMED-LESDPE-LDLDSSGEIVYAM
ZmOST1 EATIPPACSRSIN-VLADGLDMDDDMD-DLDSDSD-LDVDSSGEIVYAM
