# Supplementary figures and images for: Characterization of thiol‐based redox modifications of Brassica napus SNF1‐related protein kinase 2.6‐2C
Source: FEBS Open Bio. 2018 Mar 5;8(4):628–45. doi: 10.1002/2211-5463.12401 (PMC5881534; doi:10.1002/2211-5463.12401)

(A)

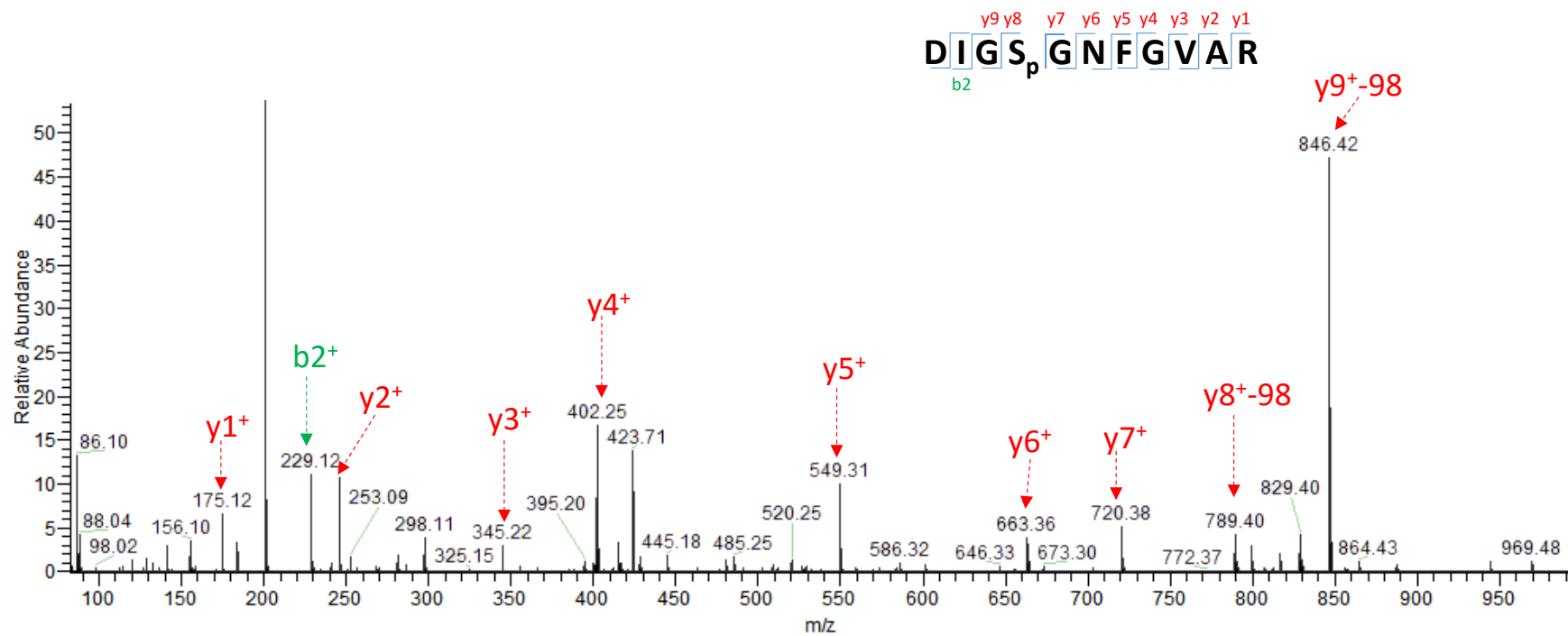

(B)

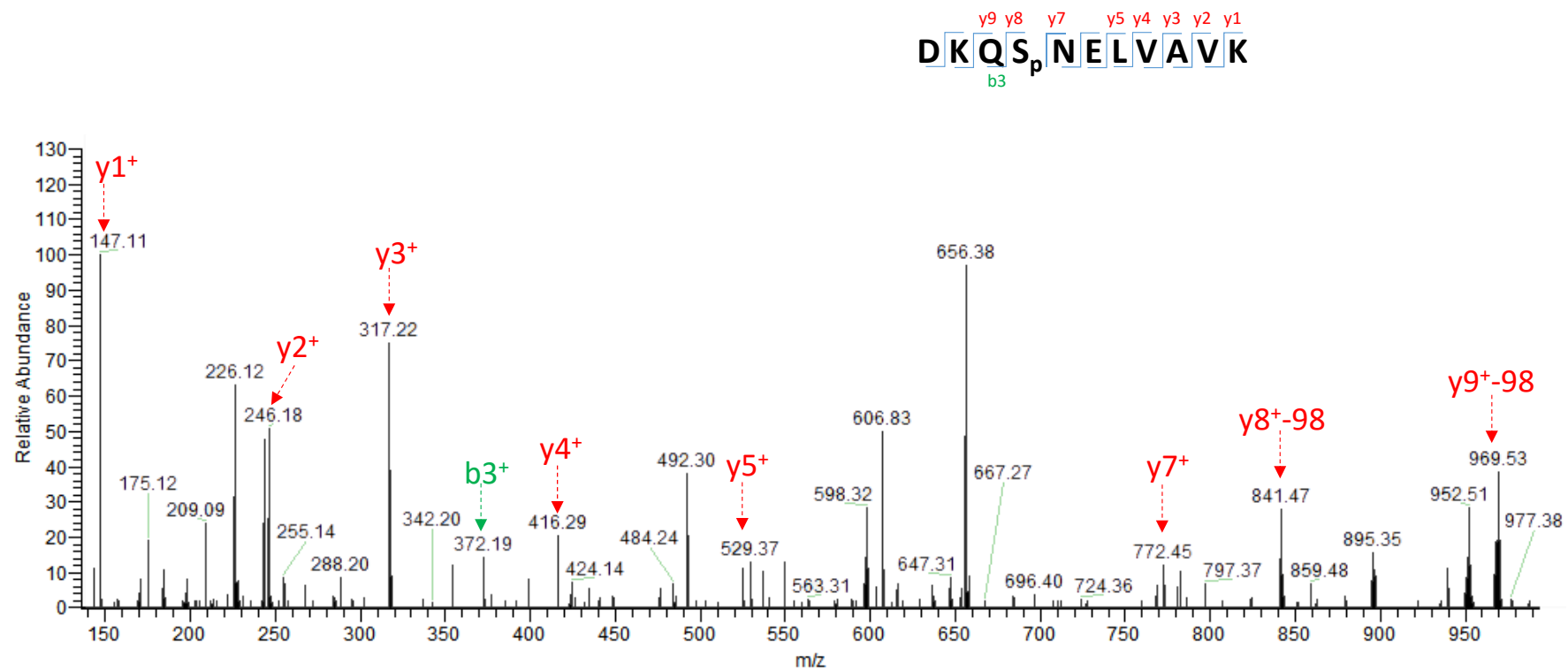

(C)

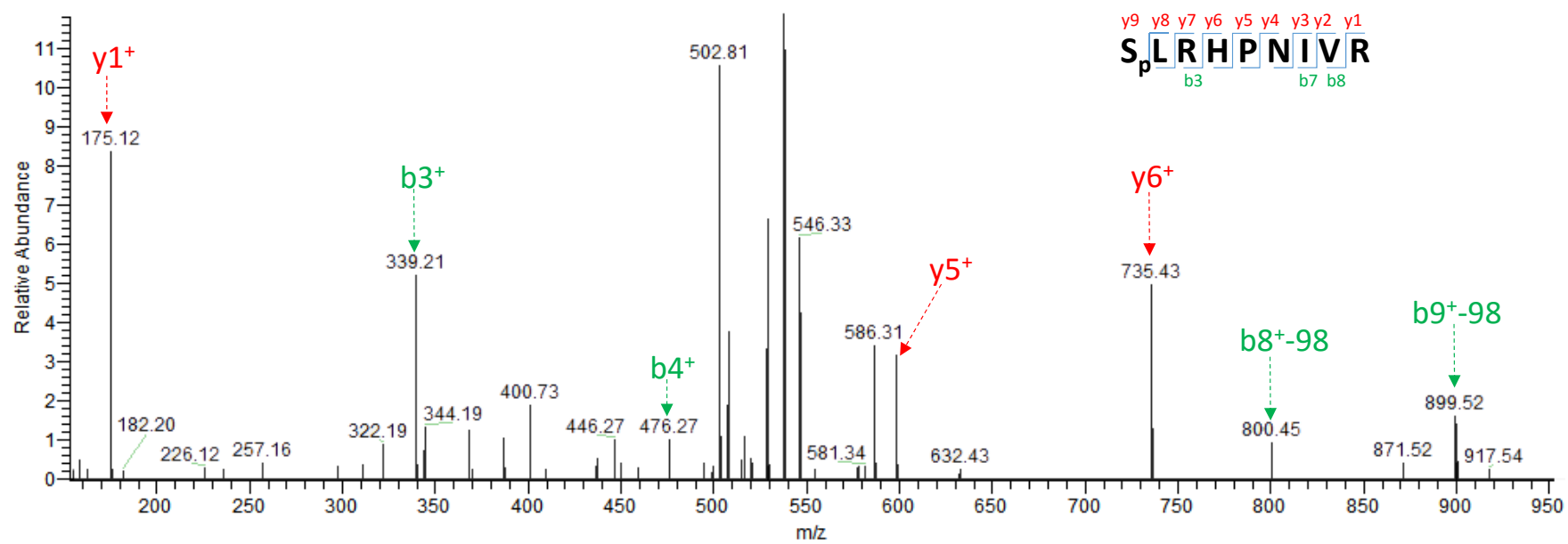

(D)

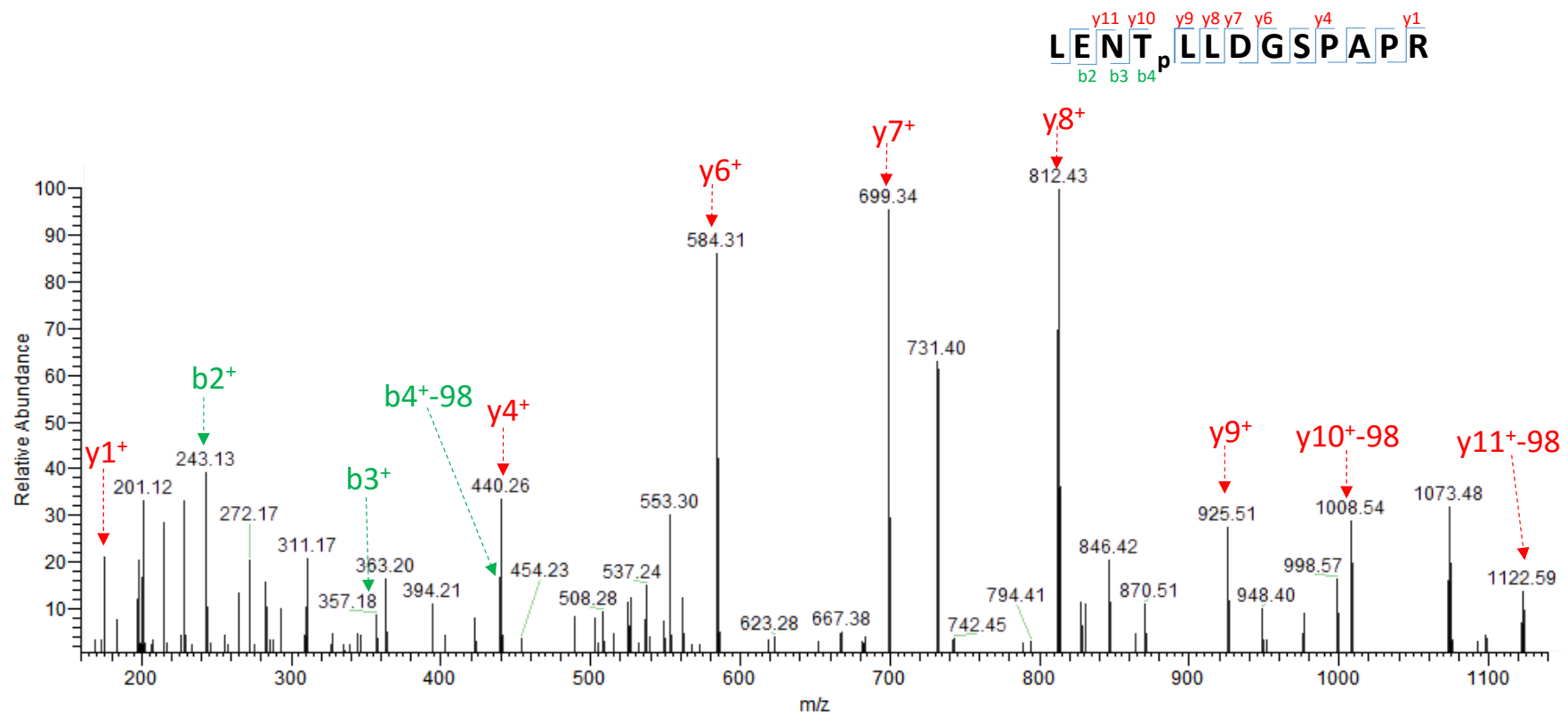

(E)

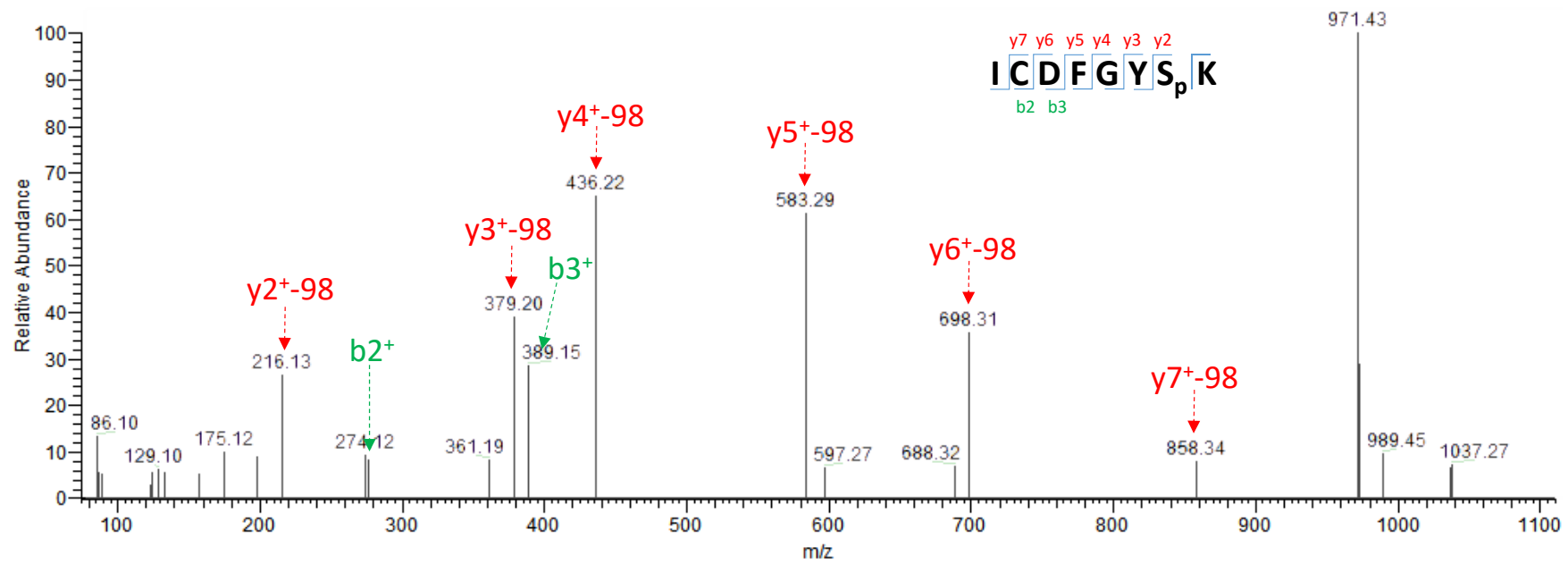

(F)

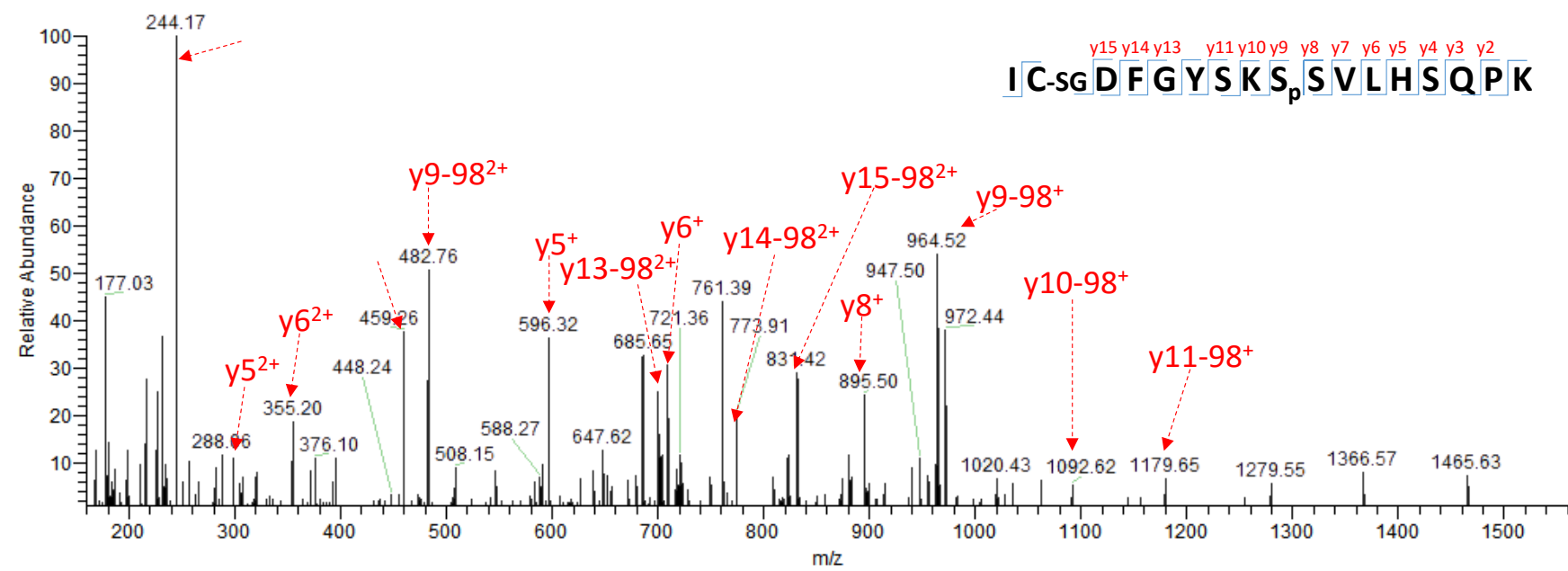

(G)

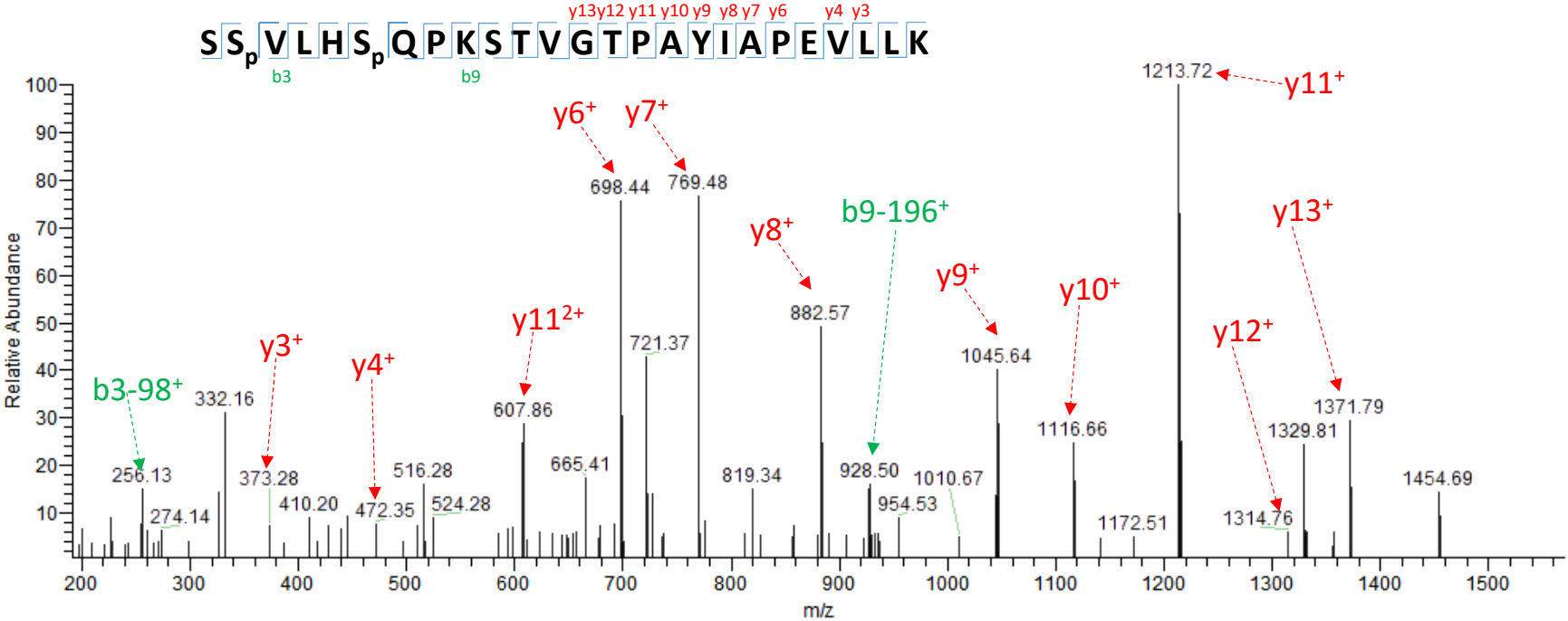

(H)

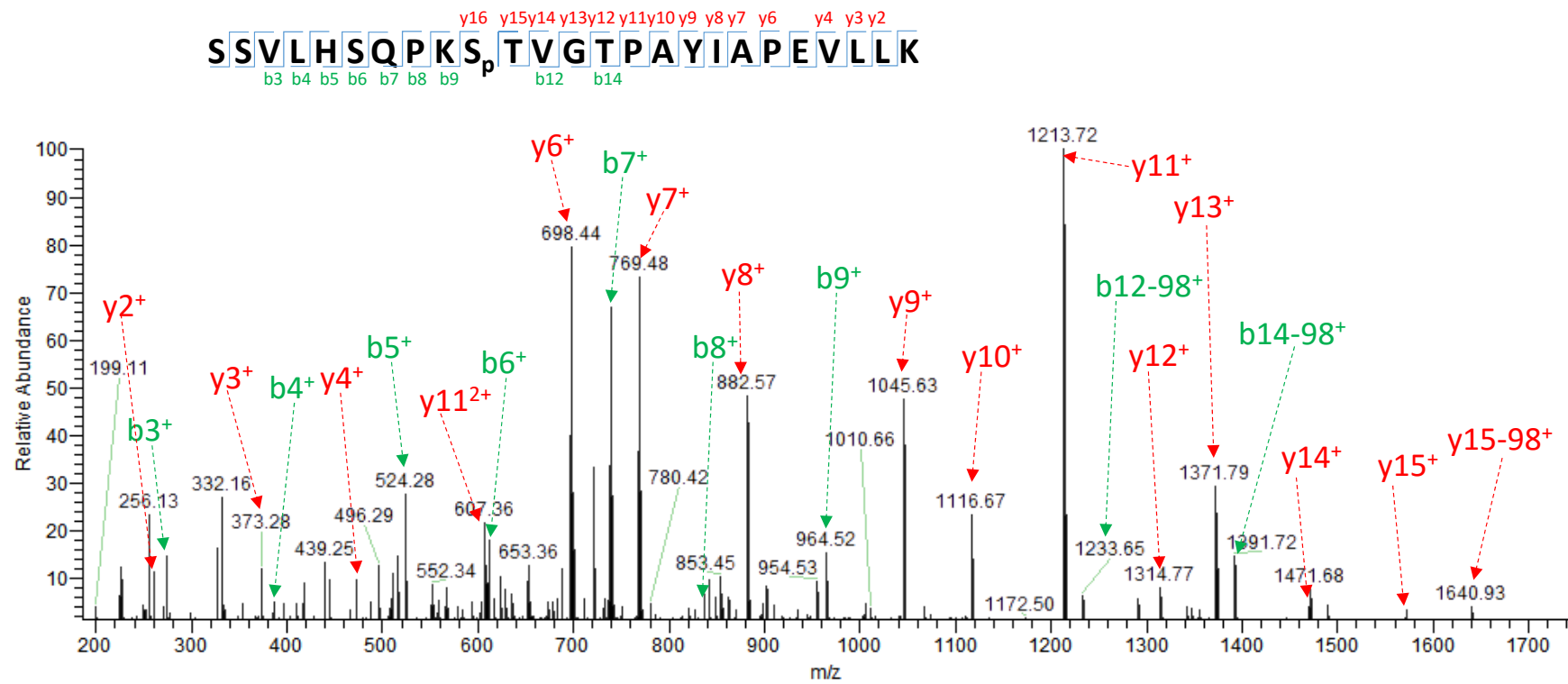

(I)

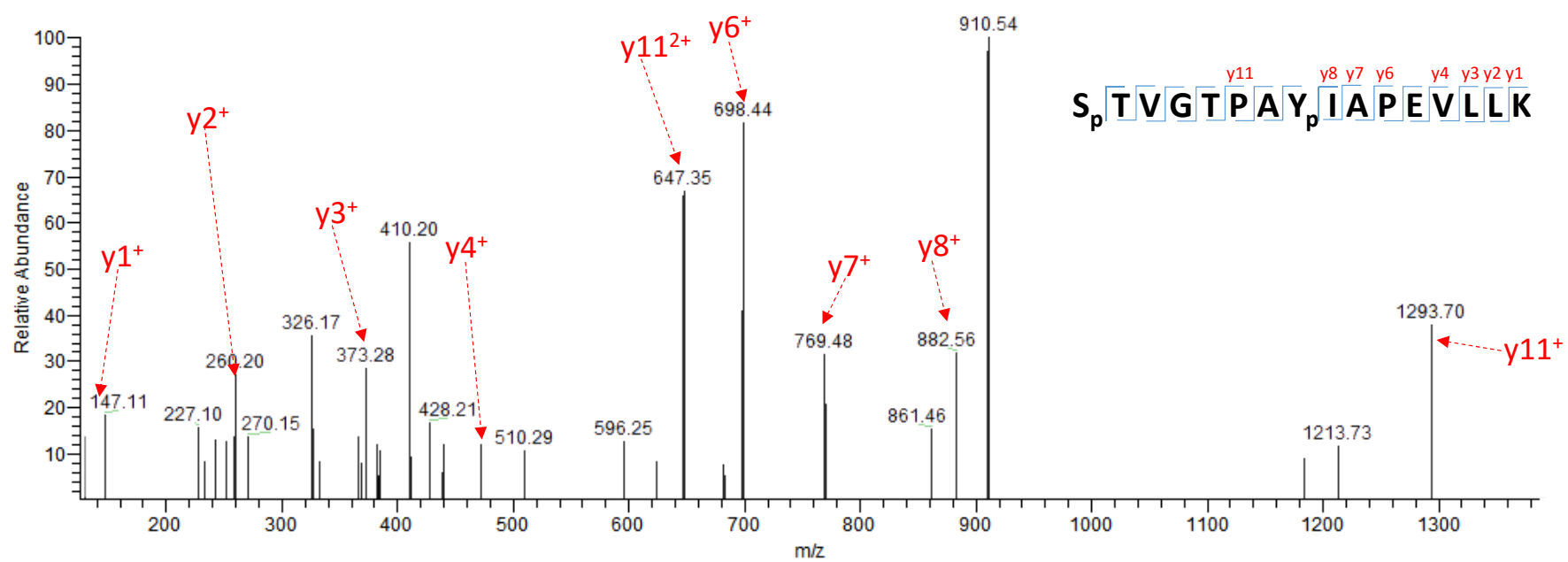

(J)

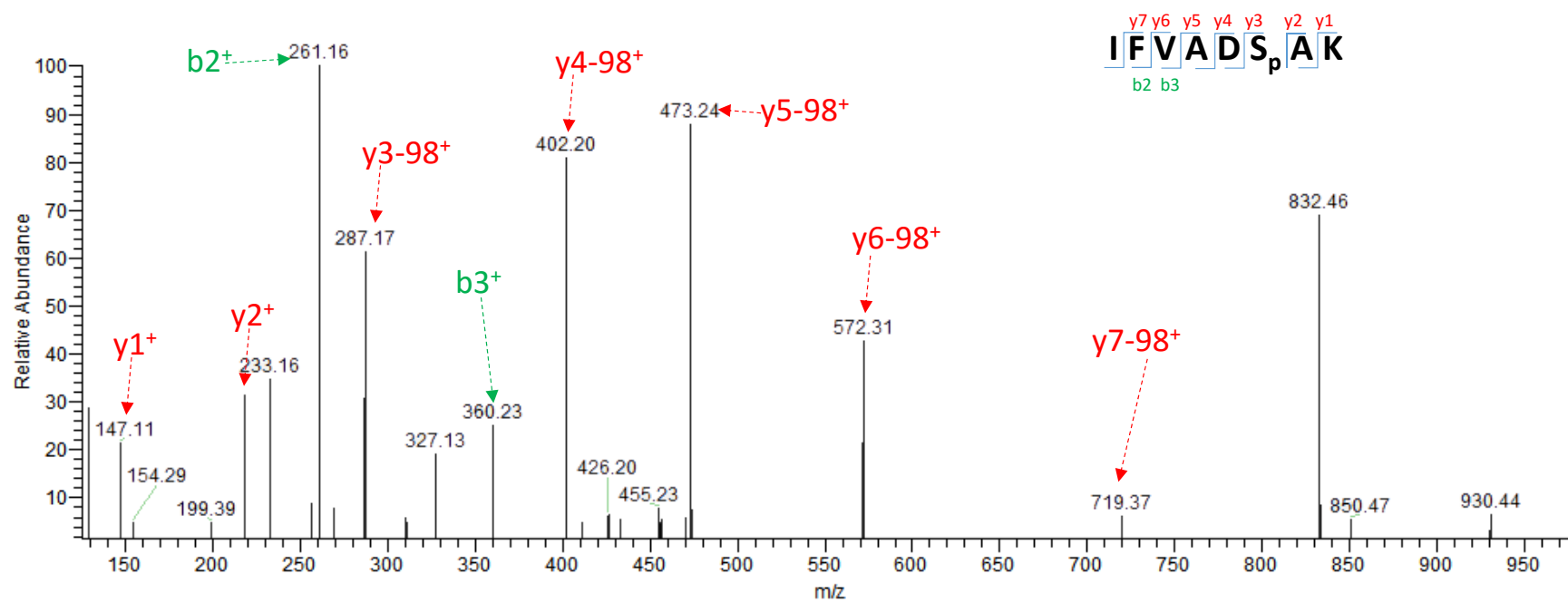

(K)

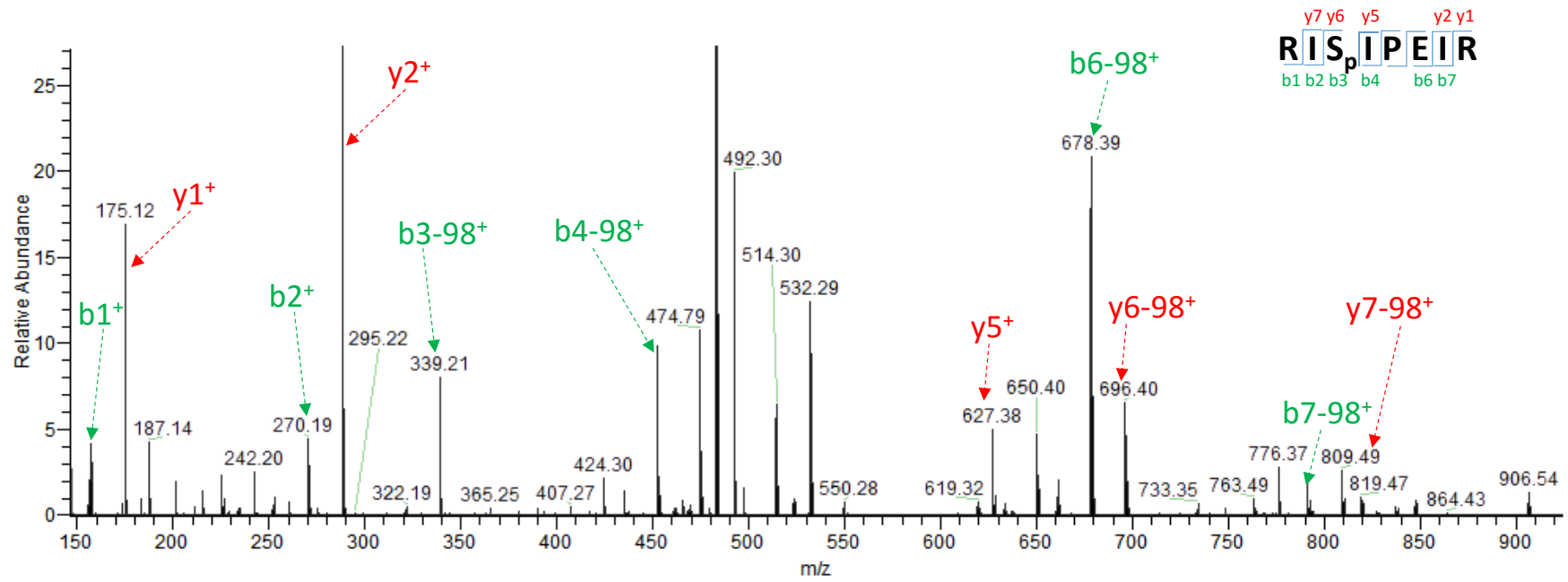

Supplement: Supplementary file 2 — Fig. S2. Phosphorylation sites identification of BnSnRK2.6‐2C by mass spectrometry. MS/MS spectra of peptides containing phosphorylated S29 (A), S43 (B), S71 (C), T146 (D), S164 (E), S166 (F), S167 and S171 (G), S175 (H,I), Y182 (I), S262 (J), and S267 (K) of BnSnRK2.6‐2C detected by LC‐MS/MS. MS/MS ions used for peptides identification were labeled. [file FEB4-8-628-s002.pdf]

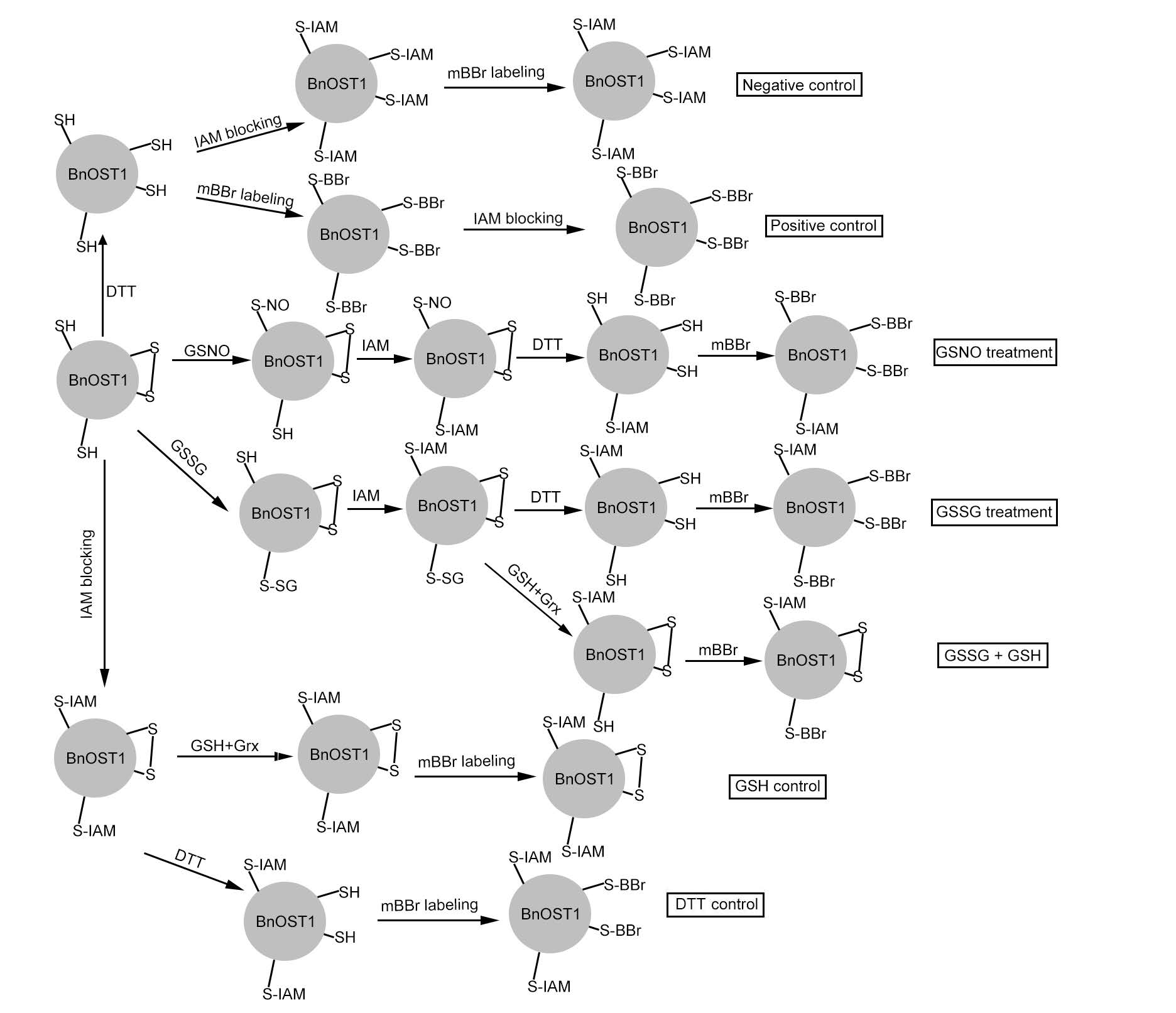

Supplement: Supplementary file 3 — Fig. S3. Diagram depicting monobromobimane (mBBr) labeling workflow to identify reversible oxidations of cysteine residues in BnSnRK2.6‐2C and the positive and negative controls (Table1for results). Positive control: The protein was reduced with DTT, labeled with mBBr directly, and then treated with IAM. Negative control: DTT‐reduced free thiols were blocked with IAM before the mBBr labeling. DTT control: The protein was blocked with IAM, then reduced with DTT and labeled with mBBr. GSH control: The protein was blocked with IAM, and then reduced with GSH and Grx, followed by mBBr labeling. GSNO, GSSG and GSSG + GSH treatments (reverse labeling): After the treatment, IAM was used to block the remaining free thiol groups. Reversibly oxidized cysteine residues were then reduced with DTT or GSH and Grx, followed by mBBr labeling. [file FEB4-8-628-s003.jpg]

(A)

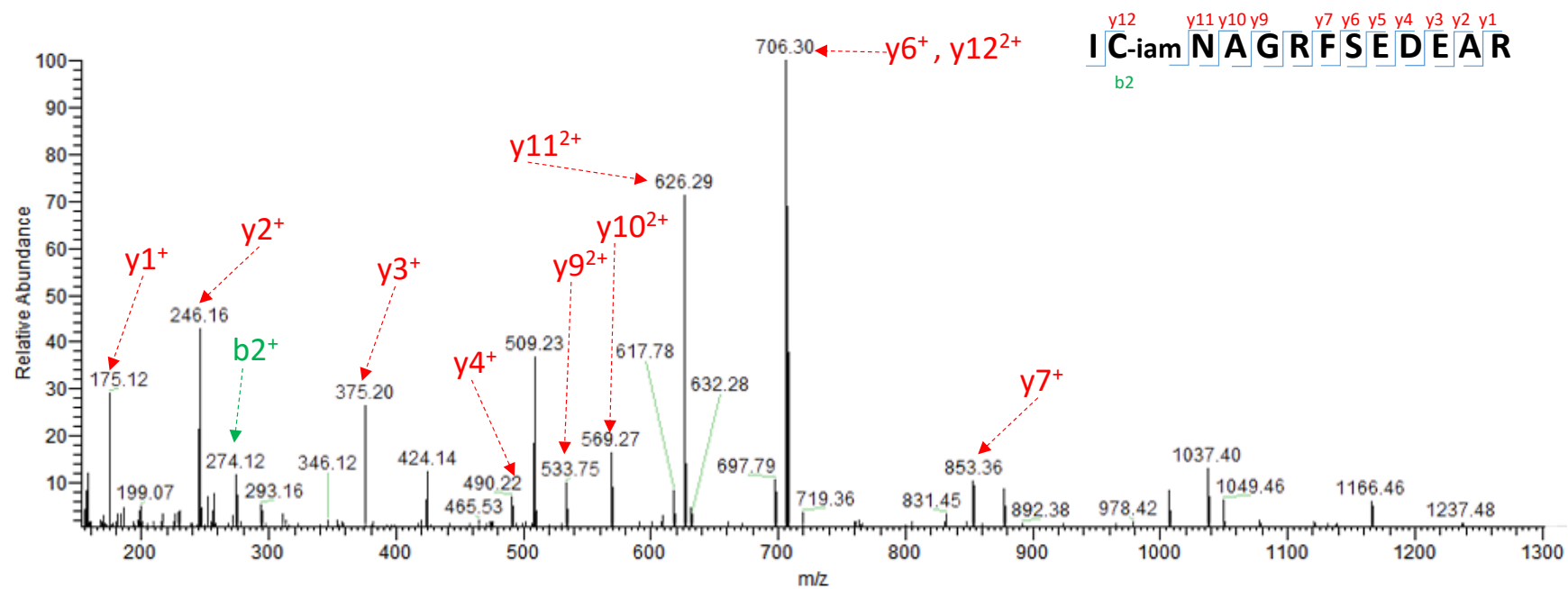

(B)

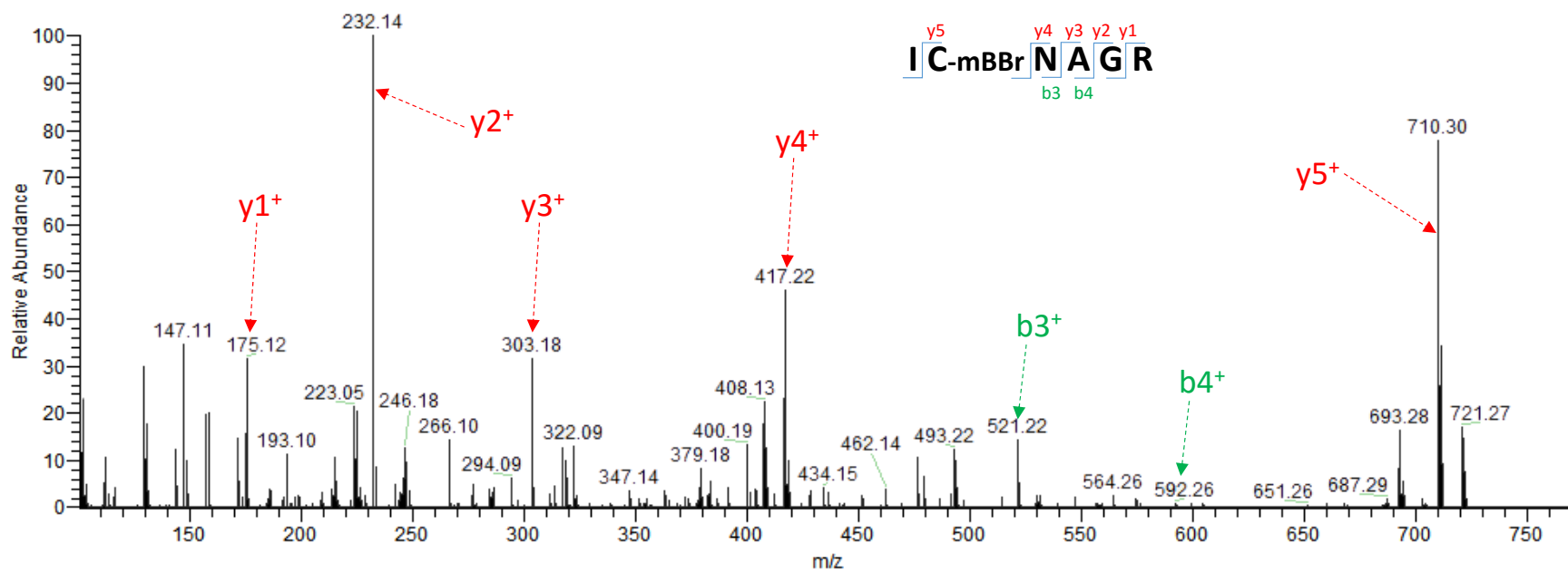

(C)

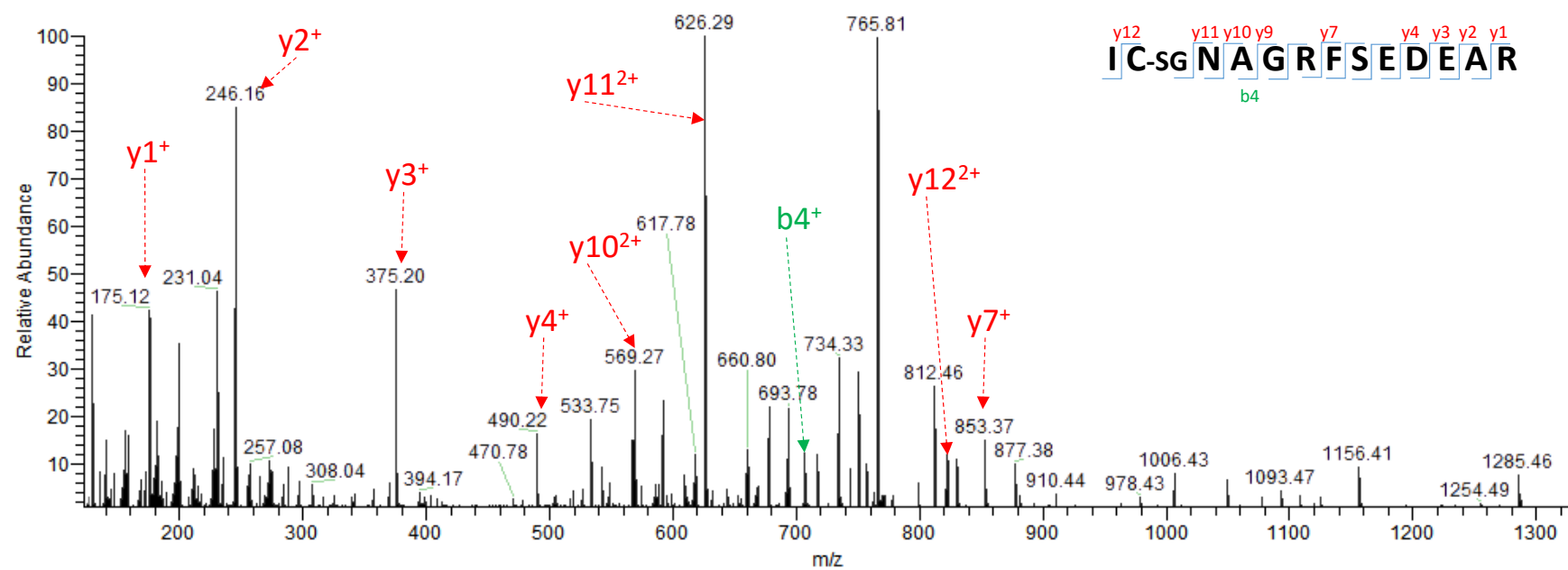

(D)

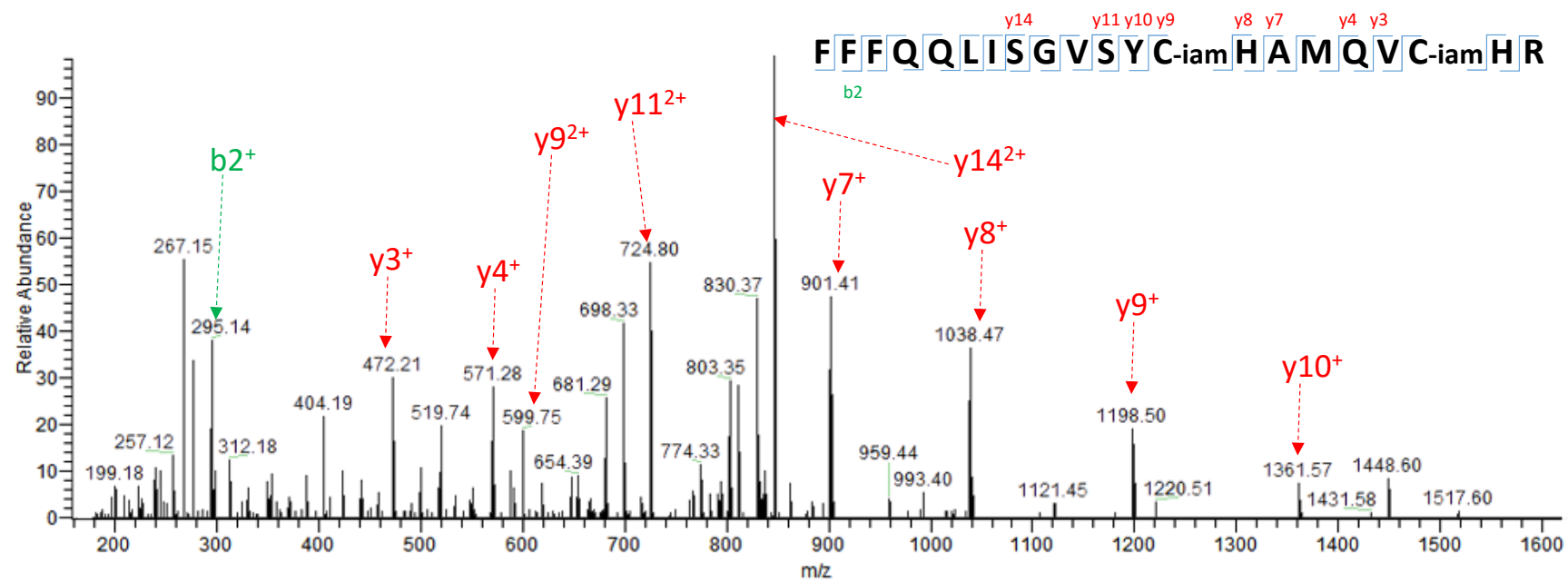

(E)

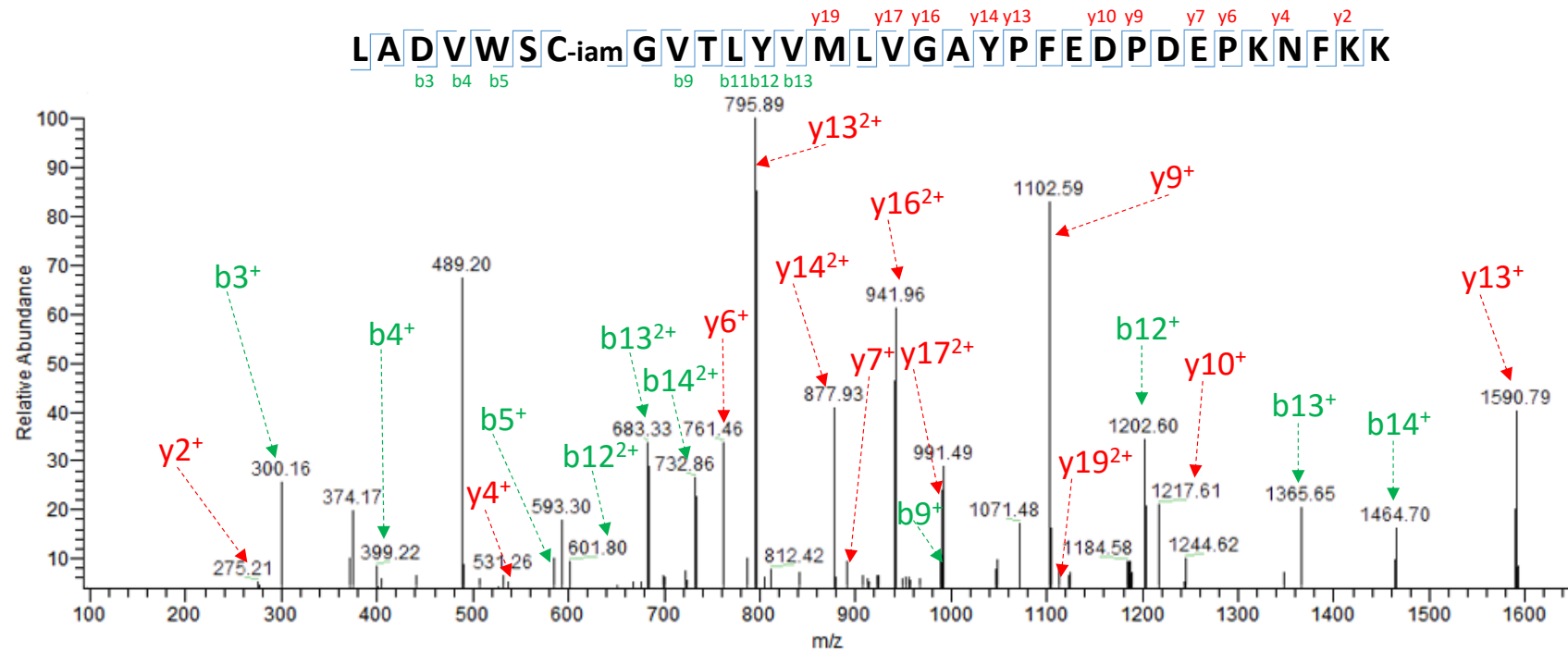

(F)

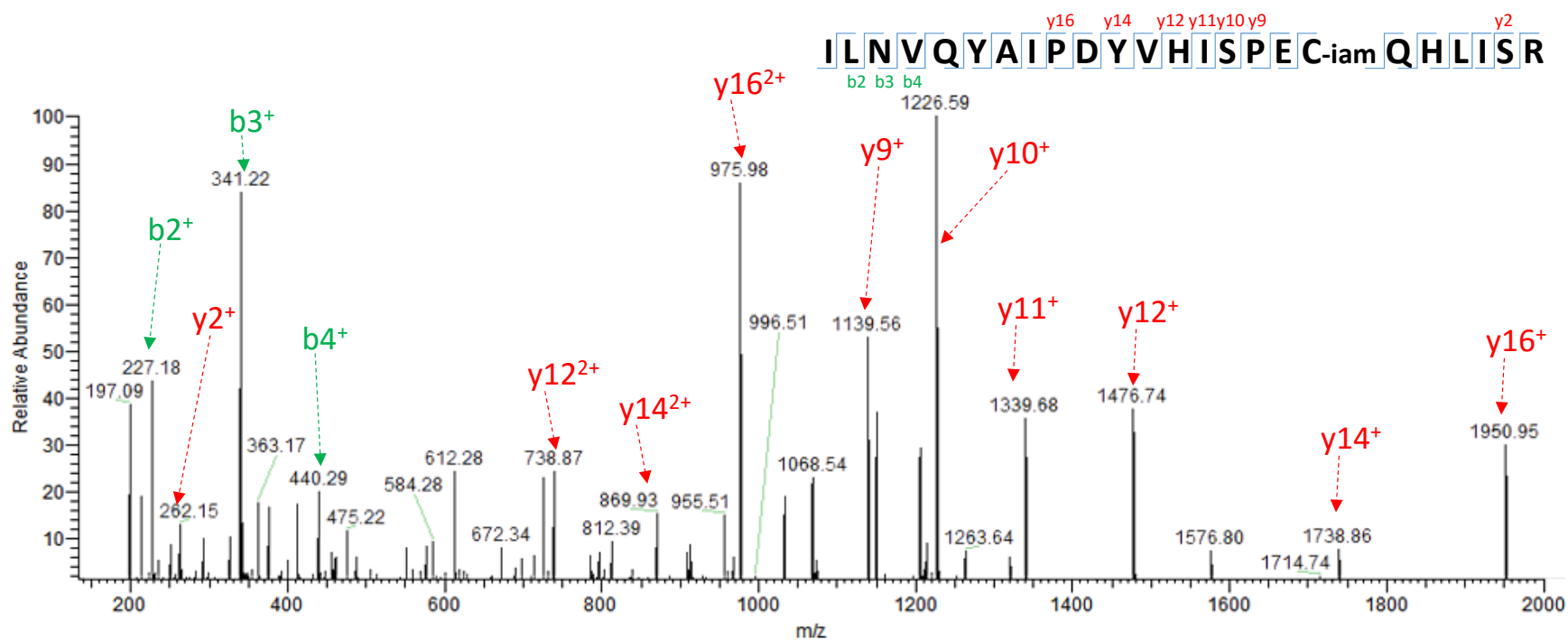

(G)

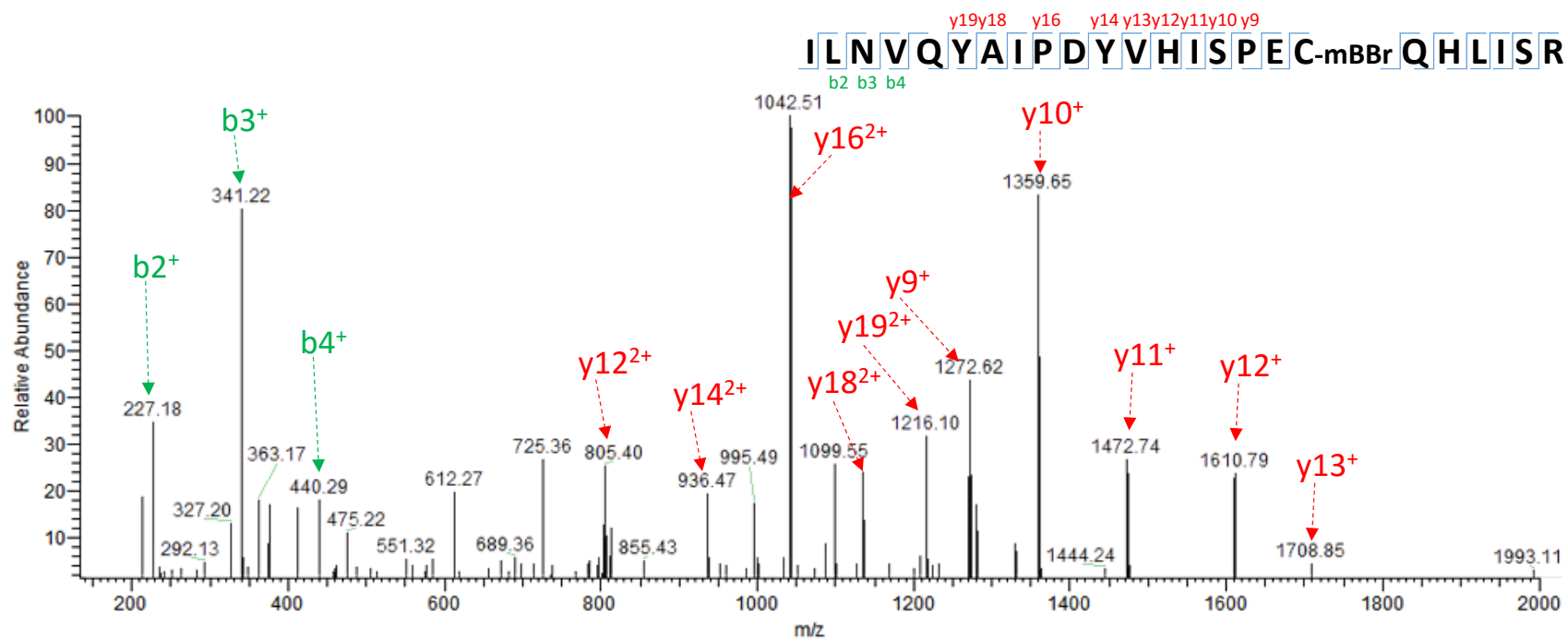

Supplement: Supplementary file 4 — Fig. S4. Identification of BnSnRK2.6‐2C cysteine residue modifications in response to GSNO and GSSG treatments by mass spectrometry. MS/MS spectra of peptides containing C107 modified by IAM (A), mBBr (B), or glutathione group (C), C131 and C137 modified by IAM (D), C203 modified by IAM (E), C250 modified by IAM (F) or mBBr (G) in BnSnRK2.6‐2C by LC‐MS/MS. MS/MS ions used for peptides identification were labeled. [file FEB4-8-628-s004.pdf]

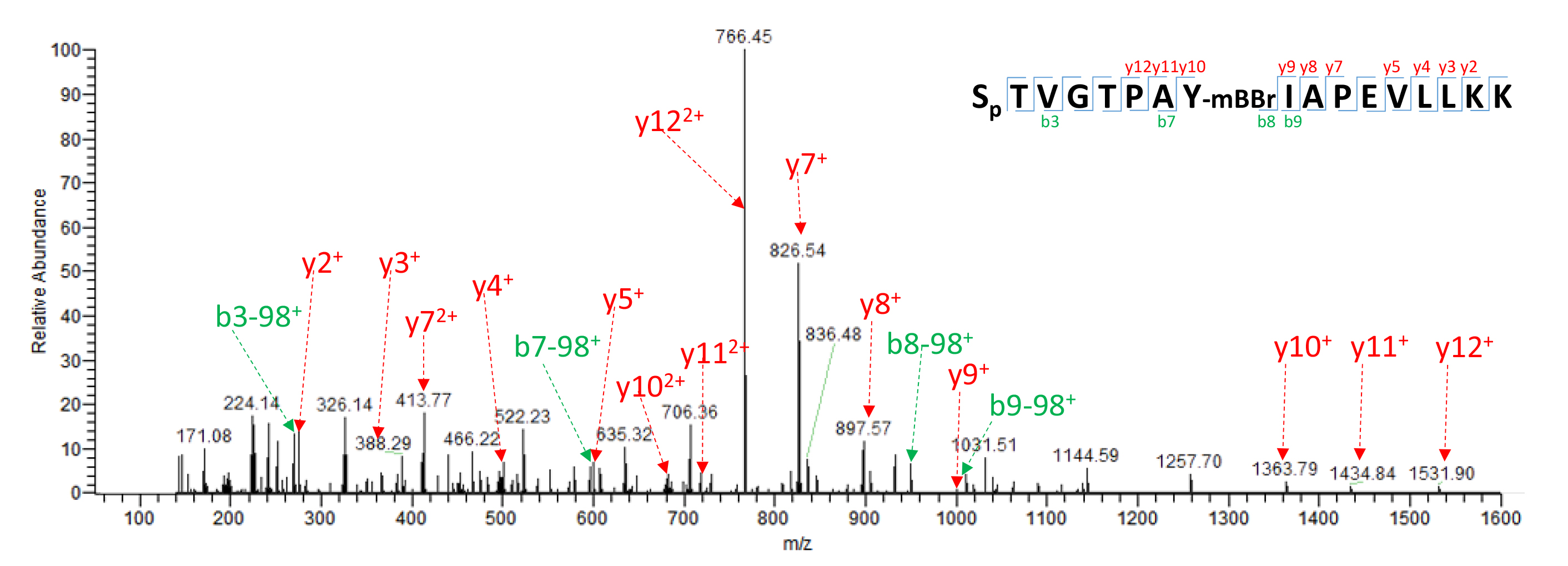

Supplement: Supplementary file 5 — Fig. S5. Identification of peptides containing mBBr‐labeled Y182 in BnSnRK2.6‐2C. MS/MS ions used for peptides identification were labeled. [file FEB4-8-628-s005.jpg]
